# Heart Rate Variability (HRV) and Pulse Rate Variability (PRV) for the Assessment of Autonomic Responses

**DOI:** 10.3389/fphys.2020.00779

**Published:** 2020-07-23

**Authors:** Elisa Mejía-Mejía, Karthik Budidha, Tomas Ysehak Abay, James M. May, Panayiotis A. Kyriacou

**Affiliations:** Research Centre for Biomedical Engineering (RCBE), School of Mathematics, Engineering and Computer Science, University of London, London, United Kingdom

**Keywords:** autonomic nervous system, pulse rate variability, heart rate variability, photoplethysmography, peripheral circulation, cold stress, vasoconstriction

## Abstract

**Introduction:** Heart Rate Variability (HRV) and Pulse Rate Variability (PRV), are non-invasive techniques for monitoring changes in the cardiac cycle. Both techniques have been used for assessing the autonomic activity. Although highly correlated in healthy subjects, differences in HRV and PRV have been observed under various physiological conditions. The reasons for their disparities in assessing the degree of autonomic activity remains unknown.

**Methods:** To investigate the differences between HRV and PRV, a whole-body cold exposure (CE) study was conducted on 20 healthy volunteers (11 male and 9 female, 30.3 ± 10.4 years old), where PRV indices were measured from red photoplethysmography signals acquired from central (ear canal, ear lobe) and peripheral sites (finger and toe), and HRV indices from the ECG signal. PRV and HRV indices were used to assess the effects of CE upon the autonomic control in peripheral and core vasculature, and on the relationship between HRV and PRV. The hypotheses underlying the experiment were that PRV from central vasculature is less affected by CE than PRV from the peripheries, and that PRV from peripheral and central vasculature differ with HRV to a different extent, especially during CE.

**Results:** Most of the PRV time-domain and Poincaré plot indices increased during cold exposure. Frequency-domain parameters also showed differences except for relative-power frequency-domain parameters, which remained unchanged. HRV-derived parameters showed a similar behavior but were less affected than PRV. When PRV and HRV parameters were compared, time-domain, absolute-power frequency-domain, and non-linear indices showed differences among stages from most of the locations. Bland-Altman analysis showed that the relationship between HRV and PRV was affected by CE, and that it recovered faster in the core vasculature after CE.

**Conclusion:** PRV responds to cold exposure differently to HRV, especially in peripheral sites such as the finger and the toe, and may have different information not available in HRV due to its non-localized nature. Hence, multi-site PRV shows promise for assessing the autonomic activity on different body locations and under different circumstances, which could allow for further understanding of the localized responses of the autonomic nervous system.

## 1. Introduction

Heart rate variability (HRV) is a widely used physiological variable that non-invasively assesses the cardiac autonomic nervous system (ANS) by measuring the changes in the cardiac rhythm through time (Shaffer and Ginsberg, 2017). HRV is considered as a reflection of changes of the cardiac sympathetic and parasympathetic branches of the ANS (Clifford et al., 2006). Several models have been proposed to explain HRV (Laborde et al., 2017), which could describe the relationship between HRV, vagal tone and several physiopathological processes. Low values of HRV indices have been found to relate to cardiac events, such as myocardial infarction; progression of atherosclerosis; and heart failure (Huikuri et al., 1999). Some studies have also associated HRV values with conditions such as coronary artery disease and sudden death (Xhyheri et al., 2012), diabetes mellitus (da Silva et al., 2016), pain (Broucqsault-Dédrie et al., 2016), acute and chronic stress (Murray, 2012; Castaldo et al., 2015), metabolic syndrome (Stuckey et al., 2014), depression (Koenig et al., 2016), and bipolar disorders (Bassett, 2015). Furthermore, HRV has been used as a marker of social interaction (Shahrestani et al., 2015), sports performance (Dong, 2016; Gavrilova, 2016), and emotional states (Choi et al., 2017).

HRV information is usually measured from the electrocardiographic signal (ECG) (Clifford et al., 2006), and standards of measurement have been established in an attempt to align methodologies and allow for the comparison of results presented by different studies (Task Force of the European Society of Cardiology and The North American Society of Pacing and Electrophysiology, 1996). However, in the past few years, several studies have reported the use of physiological signals other than the ECG to extract HRV information. Hence, the term Pulse Rate Variability (PRV) has been used to refer to HRV information obtained from pulse wave signals, such as the photoplethysmograms (PPG).

Photoplethysmography (PPG) is a simple, low-cost, non-invasive, optical measurement technique which is used for the detection of blood volume changes in peripheral tissue (Kyriacou, 2006; Allen, 2007). Due to its widespread use in clinical and everyday activities, researches have tried to obtain as much information from this signal as possible. One of the main avenues which has been explored widely by researches is extraction of PRV information from PPG signals (Georgiou et al., 2018). PRV has been derived from PPG for the analysis of ANS changes under different conditions, such as the presence of mental (Dagdanpurev et al., 2018; Can et al., 2019) or somatic diseases (Bolea et al., 2017; Lan et al., 2018), during sleep (Lázaro et al., 2014; Garde et al., 2019), or for evaluating the effects of pharmacological drugs (Sluyter et al., 2016). Nonetheless, although PRV has been treated as a valid surrogate of HRV, their relationship is not entirely clear, and PRV has been found to significantly differ from HRV under certain circumstances (Schäfer and Vagedes, 2013).

It has been hypothesized that factors such as stress (Giardino et al., 2002), respiratory patterns (Jan et al., 2019), exercise (Lin et al., 2014), orthostatic changes (Pernice et al., 2018), and ambient temperature (Shin, 2016) may have different effects on PRV, when compared to HRV, and therefore affecting their relationship. However, the origin of these differences is still not clear, and may be related to changes in hemodynamics, blood pressure or pulse transit time (PTT) (Charlot et al., 2009; Gil et al., 2010; Chen et al., 2015). Since hemodynamics are largely controlled by the ANS (Fox, 2016), PRV might be affected by changes in this regulation in response to external stimuli, such as colder temperature.

Thermal balance in humans exposed to extreme weather is mainly achieved by vasoconstriction or vasodilation of vessels in the skin and peripheral tissues (Daanena and Lichtenbeltd, 2016). Specifically, cold exposure generates changes in the autonomic response of humans by activating the sympathetic nervous system to maintain homeothermy (Gordon, 2009). As explained by Gordon (2009), when exposed to cold temperatures, the thermoreceptors send this information to the hypothalamus, that regulates the rate of heat production and, if necessary, stimulates the efferent nerves in the sympathetic nervous system, which has the primary role of stimulating peripheral vasoconstriction (Mäkinen et al., 2008). This activation generates changes in cardiovascular dynamics, by changing the level of constriction of vessels, generating the involuntary contraction of muscles, and increasing heart rate, cardiac output, and blood pressure (Fox, 2016). Several techniques that reflect autonomic activity have been shown to reflect these changes during cold exposure to a different extent, such as evaluating the changes in amplitude of PPG signals (Budidha and Kyriacou, 2019), the changes in central hemodynamics variables such as augmentation index (King et al., 2013), the changes in microneurography (Sawasaki et al., 2001; Greaney et al., 2017), or the changes in HRV (Mäkinen et al., 2008; Okamoto-Mizuno, 2009; Hintsala et al., 2014).

The strength of the sympathetic vasoconstriction, however, varies between core and peripheral locations, as was verified by Budidha and Kyriacou (2019). Understanding how these changes vary in each body location could improve the comprehension of ANS activity during cold exposure and the relationship between HRV and PRV under these circumstances. Also, although HRV is mainly a reflection of vagal tone (Laborde et al., 2017), sympathetic activity may influence some of the indices obtained from HRV and PRV, and might affect PRV and HRV in a different manner when subjects are exposed to colder temperatures; and the agreement between PRV and HRV has been shown to be affected by sympathetic shift of the ANS activity (Chen et al., 2015).

To investigate the dependency of PRV on external factors such as the acquisition site and the temperature, a whole-body cold exposure study was performed on healthy volunteers. PRV and HRV information was extracted from simultaneously obtained PPG and ECG signals, respectively. Red (660 nm) PPG signals were recorded from the earlobe, the ear canal, the finger, and the toe. It was hypothesized that (1) PRV information obtained from the earlobe and ear canal might not be equally affected by cold exposure as that of the finger and the toe; and (2) the agreement between HRV and PRV is altered by whole-body cold exposure, maintaining a higher agreement between HRV and PRV measured from central locations such as the earlobe and the ear canal. The results obtained from this study are important for understanding the possible differences between HRV and PRV, and might lead to further research that aims to better understand PRV, how the sympathetic and parasympathetic activity may affect it, and its clinical applications.

## 2. Materials and Methods

### 2.1. Experimental Protocol

Twenty healthy volunteers (11 male and 9 female, 30.3 ± 10.4 years old) were recruited to take part in this study. Subjects with any cardiovascular, pulmonary, or metabolic conditions were excluded. All subjects were normotensive, normothermic, and did not take any medication at the time of the study. The study protocol was approved by Senate Research Ethics Committee at City, University of London, and all subjects gave informed consent before taking part in the study.

**Figure 1:**
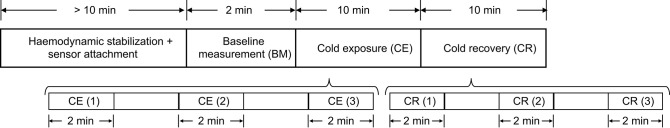
Measurement protocol for the whole-body cold exposure study.

The measurement protocol is shown in Figure 1. Subjects were asked to refrain from ingesting beverages with caffeine and alcohol, not to exercise or smoke at least 2 h before the test, and they were instructed to wear only one layer of clothes during the data acquisition period, in order to maximize the effect of the stimulus on the body. Data from all subjects was collected between 10:00 a.m. and 6:00 p.m., under controlled conditions of temperature and humidity.

Upon arrival, subjects were seated in a room maintained at 24 ± 1°C for at least 10 min, to ensure hemodynamic stabilization. After this period, the sensors for acquiring the signals were attached to the subjects. The measurement started with a 2-min baseline measurement (BM) stage, in which 2 min of signals were recorded from the subjects while the room temperature was 24 ± 1°C. The volunteers were then moved to an adjacent, temperature-controlled room, maintained at 10 ± 1°C (Cold Exposure, CE). This temperature was selected because it reflects a more realistic change in ambient temperature, which can be sustained for longer periods of time by healthy adults, and generates changes in hemodynamics (King et al., 2013). Subjects remained in this room and signals were recorded for 10 min before returning to the original room at 24 ± 1°C, for additional 10 min of signals recording (Cold Recovery, CR). During each phase of the measurement protocol, subjects were seated in a comfortable *swivel chair*, with both hands located on the arm rest.

After each of the recording on the different stages, the measurement was paused and the subject was wheeled to the room for recording the next stage. The recording was resumed as soon as the subject was moved, in order to record the shock response of the autonomic activity on the periphery.

### 2.2. Signal Acquisition and Processing

#### 2.2.1. Signal Acquisition

Disposable electrodes were placed on the left and right shoulders, and on the right hip (reference electrode) for obtaining lead I ECG signals, while PPG signals from the left index finger (F), toe (T), ear canal (EC), and earlobe (EL) were obtained from each subject during the three stages of the study. Red light (660 nm) was used for acquiring PPG's. The signal acquisition was paused during the transitions between the two rooms in order to avoid movement artifacts.

All PPG and ECG measurements were acquired using a research PPG acquisition system (*ZenPPG*) developed in the Research Center for Biomedical Engineering, at City, University of London (Budidha, 2016). All signals were acquired at a sampling rate of 1 kHz.

#### 2.2.2. PPG Signal Processing

PPG signals were down-sampled to 100 Hz to restrict the bandwidth of the signals and remove any unwanted noise. Afterwards, they were detrended, and the first and last 10 s of each stage of the protocol were removed, to eliminate any non-stationarities of the signal. Signals were then filtered using a fourth-order bandpass Butterworth filter, with cut-off frequencies of 0.1 and 2 Hz. These cutoff frequencies were selected to attenuate any unwanted noise and strengthen the pulsatile component of the PPG signal.

Different fiducial points such as systolic peaks (PKS), onsets of the pulse (ONS), maximum slope point (SLO), and the intersection point between tangent lines from the onset and the maximum slope point were obtained from each PPG signal (TI), applying an algorithm based on Li et al. (2010). Once detected, signal quality indices described in the literature (Karlen et al., 2012; Li and Clifford, 2012; Elgendi, 2016; Calle Uribe, 2018), were applied to identify the quality of the pulses segmented by each fiducial point in each PPG signal during each test stage, and those that better segmented the pulses of each PPG signal were selected and used for measuring PRV.

Using a k-means algorithm, the cardiac cycles were classified as bad and good quality. This was done assuming that during the first stage of the test the quality of the signal was maximal, and the cluster with most of the cardiac cycles of this stage was considered as the good quality (GQ) group. Hence, the cycles classified in this group during the other two stages (CE and CR) were considered as good-quality pulses. The proportion between GQ pulses and the total number of pulses was measured for each fiducial point during each stage and from each body location. Then, the fiducial point that showed the highest proportion of GQ pulses in each case were selected for further analysis. This was performed to diminish the effect of noise in the measurement of PRV, and in an attempt to automatically determine the better fiducial point for each condition, as proposed in Pinheiro et al. (2016).

#### 2.2.3. ECG Signal Processing

**Figure 2:**
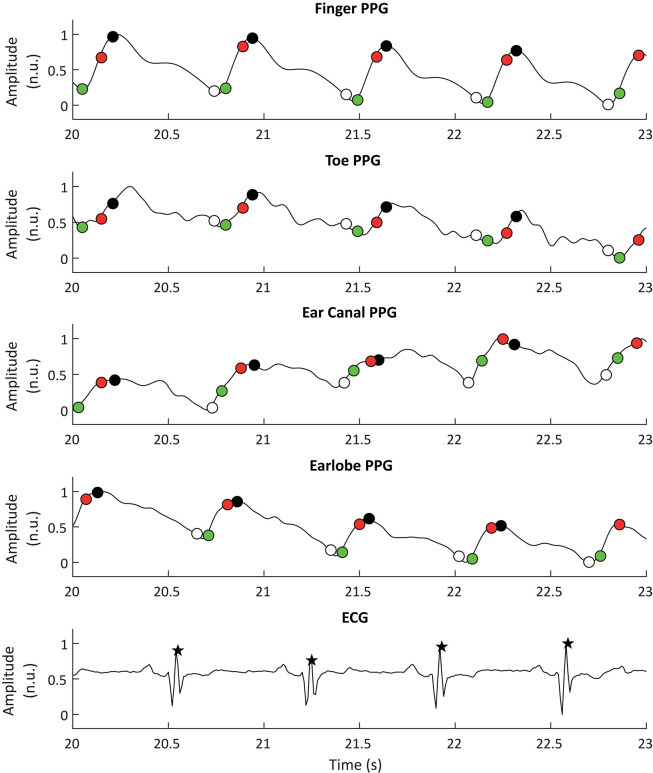
Example of photoplethysmographic (PPG) and electrocardiographic (ECG) signals used for the extraction of pulse rate variability and heart rate variability, respectively. From top to bottom, the PPG signals correspond to the signals obtained from the finger, the toe, the ear canal, and the earlobe. The black stars show the R peaks detected from the ECG signal, while the white, black, red, and green circles show the detected onsets, peaks, maximum slope points, and tangent intersection points, respectively.

ECG signals were also down-sampled to 100 Hz and R peaks were detected using an algorithm based on Pan and Tompkins (1985) and Hamilton and Tompkins (1986) algorithms. These processing steps were performed using the 2019a version of MATLAB^®^ (Mathworks, USA). Figure 2 shows a segment of PPG and ECG signals and the extracted fiducial points from each of these signals.

#### 2.2.4. HRV and PRV Analysis

Using the selected fiducial points from PPG and the R peaks obtained from the ECG, interbeat intervals (IBI's), and R-to-R intervals (RRI's) were measured for the extraction of HRV and PRV information, respectively. IBI's and RRIs that were 50% above or below their median value were corrected. Two minutes segments of IBI's and RRI's were obtained from each stage, and time- and frequency-domain indices, as well as Poincaré plot-derived indices, were obtained from these traces, as recommended in the literature (Task Force of the European Society of Cardiology and The North American Society of Pacing and Electrophysiology, 1996; Khandoker et al., 2013; Shaffer and Ginsberg, 2017).

The standard deviation of normal-to-normal intervals (SDNN), the root mean square of successive interval differences (RMSSD) and the percentage of successive intervals that differ by more than 50 ms (pNN50). For frequency-domain analysis, traces were interpolated using cubic-spline interpolation and a sampling rate of 4 Hz, and the power spectra were obtained using fast Fourier transform (FFT). The absolute and relative powers of the low-frequency (0.04–0.14 Hz, LF, and nLF) and high-frequency (0.15–0.4 Hz, HF, and nHF) bands, as well as the total power of the spectra between 0.0033 and 0.4 Hz (TP) and the ratio of the low-frequency and high-frequency powers (LF/HF) were measured. Finally, the standard deviation of data located perpendicular (SD1) and along (SD2) the line of identity of the Poincaré plot and their ratio (SD1/SD2) were obtained (Khandoker et al., 2013).

As explained by Shaffer and Ginsberg (2017), SDNN reflects both the SNS and PNS activity, although it is thought that its main source of variation in short-term recordings is the respiratory sinus arrhythmia (RSA), while RMSSD is the primary time-domain measure to evaluate the vagal activity reflected in HRV; pNN50 has been shown to be closely related to PNS activity and RMSSD measurements. Similarly, these authors explain that LF is mainly produced by both SNS and PNS activity, together with blood pressure regulation performed by baroreceptors (Shaffer and Ginsberg, 2017); during normal respiratory rates, this frequency band is thought to reflect baroreflex activity and not cardiac sympathetic innervation, whereas during slow breathing, LF can be modified by vagal activity (Khandoker et al., 2013; Shaffer and Ginsberg, 2017). HF, on the other hand, is considered as the respiratory band and reflects mainly parasympathetic activity and RSA; it has been observed that total vagal blockade eliminates most of the frequency components in this frequency band (Pomeranz et al., 1985; Malliani et al., 1991). For both LF and HF bands, it is possible to obtain a measure of relative power in normalized units, which emphasizes the behavior of cardiac autonomic activity (Task Force of the European Society of Cardiology and The North American Society of Pacing and Electrophysiology, 1996). Also, TP is the summation of both LF and HF bands (Task Force of the European Society of Cardiology and The North American Society of Pacing and Electrophysiology, 1996). LF/HF is a more controversial measurement, traditionally thought to reflect the sympathovagal balance, especially in studies involving 24-h recordings (Shaffer and Ginsberg, 2017); however, this fact has been questioned due to the important effect that PNS activity has on LF as well as the lack of correlation between increased sympathetic activity and higher values of this ratio (Billman, 2013). Hence, LF/HF should not be considered as a marker of sympathovagal balance but as a reflection of baroreflex activity (Goldstein et al., 2011).

Finally, Poincaré plot-derived indices have also been associated with changes in the SNS and PNS activity. As explained by Khandoker et al. (2013), the dispersion of the points perpendicular to the line of identity, i.e., SD1, reflects the short-term variability of interbeat intervals, and relates to RMSSD; whereas the dispersion of the points along the line of identity, i.e., SD2, relates to the standard deviation of the interbeat intervals, the SDNN index.

### 2.3. Statistical Analysis

The aim of this study was to compare the behavior of core and peripheral PRV during cold exposure, and to evaluate if and how PRV differed to HRV during mild whole body cold exposure. Hence, two hypotheses were proposed: (1) PRV from core vasculature (ear canal and earlobe) is less affected by cold exposure than PRV from the periphery (finger and toe); and (2) PRV from core vasculature is more similar to HRV than PRV from peripheral tissue, especially during cold exposure.

To evaluate the first hypothesis, PRV indices obtained during the first 2 min of each stage of the test were compared using repeated-measures analysis of variance (ANOVA), with sphericity corrections. Multiple comparisons with pairwise t-tests and Bonferroni corrections were performed in case the ANOVA showed a statistically significant difference during at least one stage. Also, the first (min 0–2), middle (min 4–6), and last (min 8–10) segments of cold exposure and cold recovery stages were compared to baseline measurement, to evaluate the behavior of PRV and HRV indices when the ambient temperature was changing and during stabilization in each stage.

The second hypothesis was evaluated using Bland-Altman analysis, to assess the agreement between PRV and HRV indices during the first 2 min of each stage of the test. A Bland-Altman ratio (BAR) was defined as the ratio of half the range of limits of agreement (LoA, Equation 1) to the average of the pairwise measurement means, as proposed by Peng et al. (2015) (Equation 2). Agreements were considered as good (BAR ≤ 10%), moderate (10% ≤ BAR ≤ 20%), or insufficient (BAR ≥ 20%). Also, the behavior of the indices extracted from PRV and HRV during each stage of the test was evaluated using a Friedman rank sum test and *post hoc* analyses were performed using Nemenyi's test. Finally, the level of linear relationship between the indices, was assessed using Pearson or Spearman correlation analysis, for normally and non-normally distributed data respectively. Normality of data was determined using a Shapiro-Wilk test and a significance level of 5% (*p*-value < 0.05) was considered significant for all analyses.

(1) $$\begin{array}{l} LoA=\bar{\left(x\right)}\pm 1.96\sigma_{x},x=HRV-PRV \end{array}$$

(2) $$\begin{array}{l} BAR=|\frac{1.96\left(\sigma_{x}\right)}{\bar{\left(HRV+PRV\right)}}|,x=HRV-PRV \end{array}$$

## 3. Results

### 3.1. Selection of Fiducial Points

After applying the proposed algorithm for selecting the best fiducial point in each condition, it was observed that the lower proportion of good quality cardiac cycles was obtained in the finger and toe, i.e., the peripheral tissue. The most accurate results were obtained when cardiac cycles were segmented using the intersection of tangent lines (TI) and the location of the maximum slope (SLO) as fiducial points. The lowest performance was achieved when the systolic peaks (PKS) were used, except for the finger PPG in which the performance of the peak detection algorithm was better than most of the others. A summary of the behavior of the extracted indices from these fiducial points and from HRV is shown in Table 1.

**Table 1:** Mean value ± standard deviation of indices measured from HRV and PRV data.

**Stage**	**Index**	**HRV**	**PRV**			
			**Finger**	**Toe**	**Ear canal**	**Earlobe**
BM	SDNN (s)	0.048 ± 0.02	0.057 ± 0.02	0.095 ± 0.07	0.109 ± 0.12	0.059 ± 0.02
	RMSSD (s)	0.045 ± 0.02	0.075 ± 0.02	0.134 ± 0.10	0.156 ± 0.18	0.076 ± 0.03
	pNN50	0.258 ± 0.20	0.339 ± 0.14	0.478 ± 0.19	0.464 ± 0.19	0.343 ± 0.16
	LF (s^2^)	527.5 ± 420.1	498.4 ± 244.6	3209.5 ± 6820.0	8910.4 ± 29673.2	534.4 ± 251.6
	HF (s^2^)	907.5 ± 928.3	1195.9 ± 742.0	4716.0 ± 6859.3	4399.0 ± 6505.8	1246.2 ± 835.5
	TP (s^2^)	2024.7 ± 1487.0	2103.6 ± 1149.5	8525.9 ± 13472.2	15964.3 ± 43563.8	2151.1 ± 1173.5
	nLF (n.u.)	0.293 ± 0.15	0.268 ± 0.11	0.276 ± 0.13	0.324 ± 0.13	0.289 ± 0.13
	nHF (n.u)	0.398 ± 0.14	0.555 ± 0.08	0.568 ± 0.12	0.527 ± 0.16	0.549 ± 0.13
	LF/HF	0.947 ± 0.85	0.506 ± 0.26	0.554 ± 0.42	0.887 ± 1.22	0.604 ± 0.48
	SD1 (s)	0.032 ± 0.01	0.053 ± 0.02	0.094 ± 0.07	0.111 ± 0.13	0.054 ± 0.02
	SD2 (s)	2.528 ± 0.35	2.524 ± 0.36	2.782 ± 0.74	3.057 ± 1.56	2.536 ± 0.35
	SD1/SD2	0.013 ± 0.01	0.021 ± 0.01	0.032 ± 0.02	0.031 ± 0.02	0.021 ± 0.01
CE	SDNN (s)	0.066 ± 0.03	0.075 ± 0.03	0.135 ± 0.07	0.134 ± 0.08	0.073 ± 0.03
	RMSSD (s)	0.066 ± 0.04	0.099 ± 0.04	0.193 ± 0.10	0.184 ± 0.11	0.095 ± 0.04
	pNN50	0.396 ± 0.25	0.409 ± 0.16	0.623 ± 0.17	0.520 ± 0.15	0.478 ± 0.16
	LF (s^2^)	1228.3 ± 1563.1	1450.3 ± 1417.9	5258.6 ± 7295.6	7957.4 ± 15225.5	1074.6 ± 799.8
	HF (s^2^)	2380.1 ± 3516.1	2543.3 ± 2246.4	9538.4 ± 7976.8	7727.5 ± 8258.2	2592.9 ± 2312.8
	TP (s^2^)	4139.3 ± 5155.4	4528.8 ± 3885.1	16342.6 ± 16589.6	18075.5 ± 23059.4	4129.6 ± 3175.1
	nLF (n.u.)	0.307 ± 0.17	0.315 ± 0.12	0.278 ± 0.12	0.320 ± 0.15	0.268 ± 0.11
	nHF (n.u.)	0.498 ± 0.21	0.553 ± 0.12	0.626 ± 0.16	0.571 ± 0.17	0.596 ± 0.13
	LF/HF	0.924 ± 0.93	0.651 ± 0.44	0.530 ± 0.38	1.388 ± 3.73	0.506 ± 0.33
	SD1 (s)	0.047 ± 0.03	0.070 ± 0.03	0.136 ± 0.07	0.130 ± 0.08	0.067 ± 0.03
	SD2 (s)	2.613 ± 0.35	2.638 ± 0.38	3.050 ± 0.82	3.449 ± 1.84	2.626 ± 0.36
	SD1/SD2	0.018 ± 0.01	0.026 ± 0.01	0.043 ± 0.02	0.037 ± 0.01	0.026 ± 0.01
CR	SDNN (s)	0.057 ± 0.02	0.074 ± 0.03	0.127 ± 0.06	0.110 ± 0.08	0.066 ± 0.02
	RMSSD (s)	0.060 ± 0.03	0.100 ± 0.05	0.180 ± 0.08	0.154 ± 0.10	0.086 ± 0.03
	pNN50	0.326 ± 0.25	0.448 ± 0.19	0.588 ± 0.14	0.513 ± 0.20	0.372 ± 0.15
	LF (s^2^)	697.0 ± 603.0	1003.7 ± 904.1	5003.6 ± 6751.8	6446.5 ± 13699.1	870.7 ± 687.1
	HF (s^2^)	1586.7 ± 2052.1	2592.7 ± 2455.7	8123.6 ± 6813.6	5684.9 ± 6215.7	1996.8 ± 1422.7
	TP (s^2^)	2938.5 ± 2871.7	4182.9 ± 3601.2	14634.3 ± 14235.6	13936.7 ± 22646.1	3376.0 ± 2299.0
	nLF (n.u.)	0.273 ± 0.15	0.250 ± 0.09	0.291 ± 0.10	0.303 ± 0.14	0.250 ± 0.09
	nHF (n.u.)	0.473 ± 0.23	0.583 ± 0.16	0.584 ± 0.13	0.545 ± 0.19	0.587 ± 0.15
	LF/HF	1.098 ± 1.23	0.501 ± 0.30	0.547 ± 0.31	0.752 ± 0.74	0.476 ± 0.26
	SD1 (s)	0.042 ± 0.02	0.071 ± 0.03	0.127 ± 0.06	0.109 ± 0.07	0.061 ± 0.02
	SD2 (s)	2.632 ± 0.36	2.632 ± 0.35	3.075 ± 0.86	3.145 ± 1.25	2.643 ± 0.35
	SD1/SD2	0.016 ± 0.01	0.027 ± 0.01	0.040 ± 0.01	0.033 ± 0.01	0.023 ± 0.01

*BM, baseline measurement; CE, cold exposure; CR, cold recovery*.

### 3.2. Changes in PRV and HRV During Cold Exposure

Results from the repeated-measures ANOVA and its related multiple comparisons for time-domain and Poincaré plot indices are shown in Table 2, while Table 3 shows the results obtained from frequency-domain indices.

**Table 2:** *P*-values obtained from the repeated-measures ANOVA and its *post-hoc* analyses, when applied to time-domain and Poincaré plot-derived indices of PRV and HRV.

**Index**	**Source**	**ANOVA**	***post-hoc* comparisons**		
			**BM vs. CE**	**CE vs. CR**	**BM vs. CR**
SDNN (s)	HRV	<0.001	0.005	0.086	0.025
	Finger PRV	0.007	0.039	1.000	0.024
	Toe PRV	<0.001	<0.001	0.295	0.003
	Ear canal PRV	0.118	–	–	–
	Earlobe PRV	0.006	0.021	0.199	0.310
RMSSD (s)	HRV	0.001	0.004	0.174	0.004
	Finger PRV	0.015	0.066	1.000	0.053
	Toe PRV	<0.001	<0.001	0.208	0.007
	Ear canal PRV	0.250	–	–	–
	Earlobe PRV	0.009	0.022	0.331	0.265
pNN50	HRV	<0.001	<0.001	0.068	0.062
	Finger PRV	0.006	0.123	0.633	0.018
	Toe PRV	<0.001	0.002	0.639	0.011
	Ear canal PRV	0.168	–	–	–
	Earlobe PRV	<0.001	<0.001	0.002	0.774
SD1 (s)	HRV	0.001	0.004	0.170	0.005
	Finger PRV	0.015	0.067	1.000	0.053
	Toe PRV	<0.001	<0.001	0.206	0.007
	Ear canal PRV	0.250	–	–	–
	Earlobe PRV	0.009	0.023	0.330	0.266
SD2 (s)	HRV	0.001	0.017	1.000	0.003
	Finger PRV	0.004	0.029	1.000	0.012
	Toe PRV	0.002	0.003	1.000	0.013
	Ear canal PRV	0.076	–	–	–
	Earlobe PRV	0.001	0.036	1.000	<0.001
SD1/SD2	HRV	0.001	0.005	0.131	0.015
	Finger PRV	0.025	0.097	1.000	0.083
	Toe PRV	0.002	0.004	0.261	0.046
	Ear canal PRV	0.044	0.178	0.060	1.000
	Earlobe PRV	0.021	0.047	0.192	0.724

*Values in red indicate statistical significance (p-value < 0.05). Sphericity corrections using Greenhouse-Geisser correction were applied when Mauchly's test showed statistically significant results, and p-values shown are after these corrections. BM, baseline measurement; CE, cold exposure; CR, cold recovery*.

**Table 3:** *P*-values obtained from the repeated-measures ANOVA and its *post-hoc* analyses, when applied to frequency-domain indices of PRV and HRV.

**Index**	**Source**	**ANOVA**	***post-hoc* comparisons**		
			**BM vs. CE**	**CE vs. CR**	**BM vs. CR**
LF (s^2^)	HRV	0.057	–	–	–
	Finger PRV	0.008	0.028	0.162	0.077
	Toe PRV	0.009	0.010	1.000	0.083
	Ear canal PRV	0.622	–	–	–
	Earlobe PRV	0.004	0.016	0.352	0.122
HF (s^2^)	HRV	0.024	0.070	0.134	0.059
	Finger PRV	0.016	0.058	1.000	0.057
	Toe PRV	<0.001	<0.001	0.236	0.013
	Ear canal PRV	0.057	–	–	–
	Earlobe PRV	0.008	0.018	0.458	0.016
TP (s^2^)	HRV	0.031	0.085	0.230	0.061
	Finger PRV	0.013	0.047	1.000	0.052
	Toe PRV	<0.001	<0.001	0.546	0.007
	Ear canal PRV	0.581	–	–	–
	Earlobe PRV	0.002	0.015	0.459	0.037

*Values in red indicate statistical significance (p-value < 0.05). Sphericity corrections using Greenhouse-Geisser correction were applied when Mauchly's test showed statistically significant results, and p-values shown are after these corrections. Normalized frequency-domain indices did not show statistical differences between stages from any of the signals. BM, baseline measurement; CE, cold exposure; CR, cold recovery*.

#### 3.2.1. Time-Domain Indices

SDNN showed statistically significant differences between baseline measurement and cold exposure when measured from any location, except for the ear canal, while RMSSD and pNN50 did not show statistically significant differences between these stages when measured from the finger and the ear canal. All indices behaved similarly when cold exposure and cold recovery were compared, except for pNN50 measured from the earlobe. Ear canal was the only location from which none of the indices measured show any statistically-significant differences among stages.

#### 3.2.2. Frequency-Domain Indices

Relative-power indices, i.e., nLF, nHF, and LF/HF, did not show any difference among stages. Regarding absolute-power indices (LF, HF, and TP), and similar to what was observed from time-domain indices, the ear canal-derived PRV indices did not show any differences among stages. HRV-derived LF and HF did not show differences among stages, and finger HF did not show differences in the *post-hoc* analyses. LF, HF, and TP did not differ between cold exposure and cold recovery when measured from any of the locations.

#### 3.2.3. Non-linear Indices

Ear canal-derived indices did not differ among stages from any of the Poincaré plot-derived indices. Similarly, SD1 and SD1/SD2 did not show differences among stages when measured from the finger. Most differences were obtained when baseline measurement and cold exposure where compared, while no differences were shown when cold exposure and cold recovery were compared.

### 3.3. Behavior of PRV and HRV Indices

**Figure 3:**
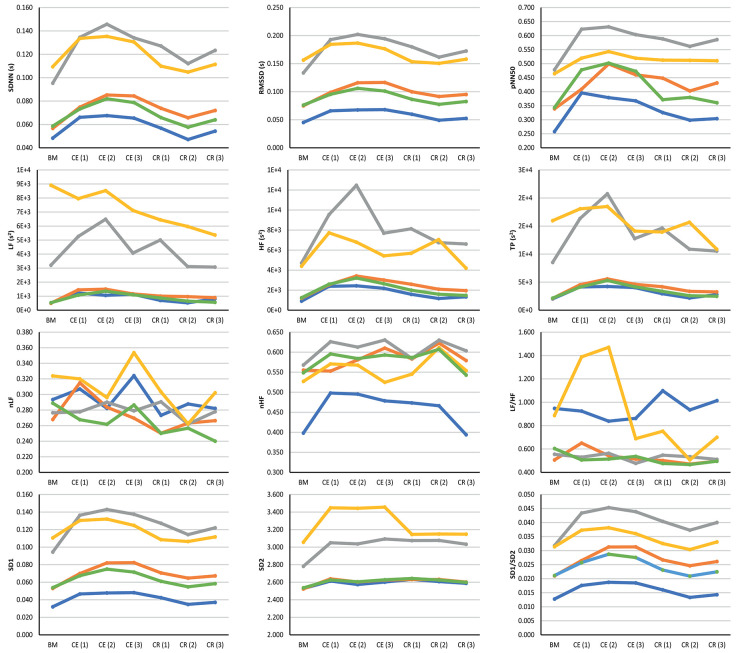
Behavior of indices measured from HRV (blue line) and PRV from the finger (orange line), toe (gray line), ear canal (yellow line), and earlobe (green line). BM: Baseline measurement; CE (1): Cold exposure between the start of the stage and the second minute of this stage; CE (2): Cold exposure between the 4th and 6th min of this stage; CE (3): Cold exposure between the 8th and 10th min of this stage; CR (1): Cold recovery between the start of the stage and the second minute of this stage; CE (2): Cold recovery between the 4th and 6th min of this stage; CE (3): Cold recovery between the 8th and 10th min of this stage.

Figure 3 illustrates the behavior of the extracted indices when 2-min segments of each stage were compared. Most indices showed a similar behavior when measured from PRV and HRV, but with a notorious overestimation of most indices when measured from PRV. LF/HF was the only index that was underestimated when measured from PRV, while nLF was the index with the least overestimation when measured from PRV. The ear canal showed the higher differences in the trends between PRV and HRV. Time-domain and non-linear indices showed that values tended to increase after baseline measurement, and then, during cold recovery, indices tended to recover to the values obtained during baseline. Frequency-domain indices did not show this behavior, probably due to the short segments used for analysis. When indices measured from the different segments were compared using a repeated-measures ANOVA (results not shown), ear canal was found to be the only location with non-statistically significant differences among each 2-min segments. Most differences observed were among baseline measurements and the segments obtained during cold exposure, and among cold exposure and cold recovery segments. On the other hand, baseline measurement and cold recovery were statistically similar, except for SD1, SD2, and RMSSD. Regarding frequency-domain indices, nLF and LF/HF failed to show any difference among stages, probably due to the short time segments used for this analysis.

### 3.4. Agreement Between HRV and PRV

#### 3.4.1. Friedman Rank Sum Tests

Results for the Friedman rank sum tests and its *post hoc* comparisons are presented in Table 4. Since the aim was to evaluate the relationship between HRV and PRV, only multiple comparisons between HRV and PRV are shown.

**Table 4:** *P*-values obtained from the Friedman rank sum test and the multiple comparison tests performed between HRV and PRV from each location (F, Finger; T, Toe; EC, Ear canal; EL, Earlobe), during each stage (BM, baseline measurement; CE, cold exposure; CR, cold recovery) and with each index.

**Stage**	**Index**	**Friedman test**	**PRV vs. HRV (Nemenyi's test)**			
			**F**	**T**	**EC**	**EL**
BM	SDNN (s)	<0.001	0.028	0.001	<0.001	0.067
	RMSSD (s)	<0.001	0.001	<0.001	<0.001	0.005
	pNN50	<0.001	0.488	<0.001	<0.001	0.429
	LF (s^2^)	<0.001	1.000	1.000	0.610	1.000
	HF (s^2^)	<0.001	0.270	0.007	0.002	0.488
	TP (s^2^)	<0.001	0.940	0.302	0.143	0.992
	nLF (n.u.)	0.527	–	–	–	–
	nHF (n.u.)	<0.001	0.013	0.001	0.007	0.016
	LF/HF	0.107	–	–	–	–
	SD1 (s)	<0.001	0.001	<0.001	<0.001	0.005
	SD2 (s)	<0.001	0.954	0.650	0.372	1.000
	SD1/SD2	<0.001	0.001	<0.001	<0.001	0.007
CE	SDNN (s)	<0.001	0.988	<0.001	<0.001	0.529
	RMSSD (s)	<0.001	0.302	<0.001	<0.001	0.028
	pNN50	<0.001	1.000	<0.001	0.061	0.429
	LF (s^2^)	<0.001	0.988	0.092	0.019	1.000
	HF (s^2^)	<0.001	0.954	<0.001	<0.001	0.569
	TP (s^2^)	<0.001	0.999	<0.001	<0.001	0.923
	nLF (n.u.)	0.558	–	–	–	–
	nHF (n.u.)	0.026	0.997	0.107	0.650	0.213
	LF/HF	0.431	–	–	–	–
	SD1 (s)	<0.001	0.302	<0.001	<0.001	0.028
	SD2 (s)	<0.001	0.988	0.005	0.014	0.960
	SD1/SD2	<0.001	0.270	<0.001	<0.001	0.013
CR	SDNN (s)	<0.001	0.372	<0.001	<0.001	0.336
	RMSSD (s)	<0.001	0.164	<0.001	<0.001	0.092
	pNN50	<0.001	0.762	0.001	0.003	1.000
	LF (s^2^)	<0.001	0.992	0.001	0.040	0.975
	HF (s^2^)	<0.001	0.610	<0.001	<0.001	0.448
	TP (s^2^)	<0.001	0.940	<0.001	0.008	0.855
	nLF (n.u.)	0.387	–	–	–	–
	nHF (n.u.)	0.160	–	–	–	–
	LF/HF	0.390	–	–	–	–
	SD1 (s)	<0.001	0.164	<0.001	<0.001	0.092
	SD2 (s)	0.001	1.000	0.143	0.187	0.827
	SD1/SD2	<0.001	0.057	<0.001	<0.001	0.107

*Values in red indicate statistically significant differences (p-value < 0.05)*.

During baseline measurement, nLF and LF/HF did not show differences between HRV and PRV, while LF, TP, and SD2 failed to show differences from *post hoc* analysis. Most of the other indices showed differences between HRV and toe PRV, and between HRV and ear canal PRV, while RMSSD, nHF, SD1, and SD1/SD2 showed differences when PRV was measured from any location.

Similar behavior was observed for nLF and LF/HF durng cold exposure. However, all other indices showed differences from *post-hoc* analyses, mainly between HRV and toe PRV, and HRV and ear canal PRV. None of the indices showed differences from all locations, but RMSSD, SD1, and SD1/SD2 showed statistically significant differences when measured from the earlobe.

Finally, during cold recovery, the same results were obtained for nLF and LF/HF. In this stage, also nHF failed to show any difference among locations, and *post hoc* analyses from SD2 did not show any differences between HRV and any of the PRV data. All differences observed were between HRV and toe PRV, and between HRV and ear canal PRV.

#### 3.4.2. Correlation Analysis

**Figure 4:**
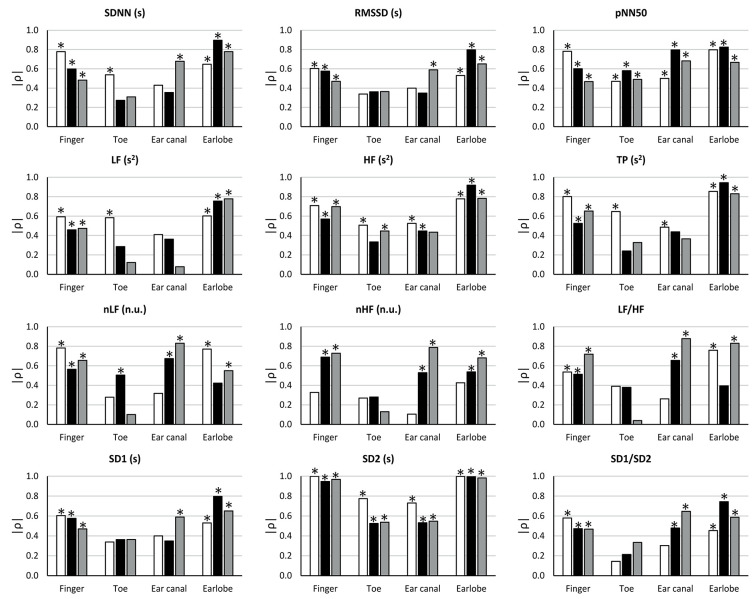
Correlation coefficients (ρ) between HRV and PRV from each location during each stage (BM: baseline measurement, white bars; CE: cold exposure, black bars; CR: cold recovery, gray bars) and with each index. Stars over bars indicate statistically significant correlations (*p*-value < 0.05).

The results from the correlation analyses between HRV and PRV are shown in Figure 4.

During baseline measurement, non-significant correlation were observed from RMSSD, nLF, LF/HF, SD1, and SD1/SD2 when these indices were measured from toe and ear canal PRV. nHF did not show significant correlations when measured from any location, and SDNN and LF had non-significant correlations when measured from the ear canal. pNN50, HF, TP, and SD2 showed statistically significant correlations when measured from all locations.

The correlation between HRV and PRV during cold exposure showed that non-significant correlations were obtained from SDNN, RMSSD, LF, TP, and SD1, when measured from the toe and the ear canal; from HF, nHF, and SD1/SD2, when measured from the toe; from nLF, when measured from the earlobe; and from LF/HF, when measured from the toe and the earlobe. Significant correlations from all locations were only observed from pNN50 and SD2.

Similarly, during cold recovery, pNN50 and SD2 showed significant correlations from all locations. However, non-significant correlations were obtained from SDNN, RMSSD, nLF, nHF, LF/HF, SD1, and SD1/SD2, when measured from the toe; from LF and TP, when measured from the toe and the ear canal; and from HF, when measured from the ear canal.

#### 3.4.3. Bland-Altman Analysis

**Figure 5:**
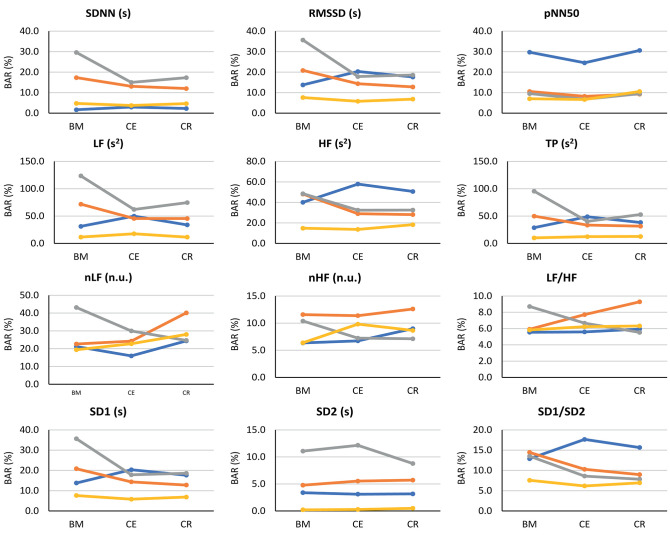
Bland-Altman ratios (BAR's) between HRV and PRV from each location (finger: blue line; toe: orange line; ear canal: gray line; earlobe: yellow line), during each stage (BM: baseline measurement; CE: cold exposure; CR: cold recovery) and with each index. Agreements were considered as good (BAR ≤ 10%), moderate (10% ≤ BAR ≤ 20%), or insufficient (BAR ≥ 20%).

Since a high correlation does not necessarily indicate a strong agreement (Bland and Altman, 1986), Bland-Altman analysis was performed to assess the agreement between PRV and HRV. Bland-Altman ratios (BAR's) are presented in Figure 5, and Bland-Altman plots are included as Supplementary Material. Agreement between HRV and PRV measured from the earlobe was the highest and most stable during the three stages, while ear canal PRV showed the worst agreement in most of the indices during the three stages.

From time-domain indices, pNN50 showed a relatively stable, moderate agreement when measured from all locations except for the finger. SDNN and RMSSD had the lowest agreement when measured from the ear canal and the toe.

Frequency-domain indices obtained using absolute powers (i.e., LF, HF, and TP) showed the worst agreement, reaching BAR's of up to 120%. Once again, earlobe showed the best agreement during the three stages. Relative-power indices had a different behavior: nHF had good agreement from most locations and stages, and moderate agreement was obtained when PRV was measured from the toe; and LF/HF showed a good agreement from every location and during all stages, and only toe-derived measurements showed a diminished agreement during cold exposure.

From Poincaré plot indices, SD2 showed good agreement in every location except for the ear canal; the earlobe, finger and toe measurements showed a good and stable agreement. SD1 showed a bad agreement from the earcanal, especially during the baseline measurement.

Bland-Altman plots showed something similar. Cold exposure affected agreement in most of the cases, but the measurement was not necessarily recovered during cold recovery. SDNN, nHF, and TP from the earlobe and the ear canal tended to recover the agreement faster during cold recovery, than that measured from the finger. However, most of the indices (i.e., nHF, LF/HF, SD1, SD2, and SD1/SD2) showed that agreement was diminished during cold exposure, but did not recover during the first 2 min of measurement during cold recovery. From these plots, it can be seen that all indices showed an overestimation of the measurement when obtained from PRV, except for LF/HF that tends to be underestimated. Over- and underestimation tend to be larger during cold exposure stage, and toe-derived PRV indices were strongly affected by under- and overestimation.

## 4. Discussion

The main aims of this study were to evaluate if PRV, understood as the time difference in pulse-to-pulse cycles measured from PPG signals, showed any difference between body locations during and after whole-body cold exposure, and if HRV and PRV differed during these thermal changes. The obtained results provide strong evidence for the primary hypotheses regarding the differences between HRV and PRV: Results indicate that cold exposure may affect PRV in different ways when obtained from peripheral and core vasculature, and that PRV may contain different information that is not available in HRV. Although HRV and PRV showed a similar trend during the whole-body cold exposure test, it was evident that PRV overestimated the indices obtained from HRV, usually in a larger scale during the cold exposure. Also, HRV and PRV should not be regarded as the same when different temperature conditions are studied, and PRV may contain different information not available from HRV, although further studies are needed to better understand the contribution of SNS to PRV measurements. These results are further discussed in the following sections.

### 4.1. Effects of Cold Exposure in Peripheral and Core Vasculature

The sympathetic control of the ANS over cutaneous blood vessels is thought to act differently over peripheral and core vasculature during cold exposure, probably caused by modifications of cutaneous blood flow to changes in temperature, which are intended to maintain thermoregulation and homeostasis (Fox, 2016). The sympathetic nervous system generates vasoconstriction in the cutaneous vessels when the temperature is low, producing a decrease in the cutaneous blood flow, which reduces the rate at which the body losses heat, and the amount of blood that is traveling to peripheral tissues such as the fingertips, the palms of the hands, the toes, and the nose, among others.

Budidha and Kyriacou (2019) and Alian et al. (2011a,b) have reported differences in core and peripheral vasculature response to cardiovascular changes. The former showed that PPG amplitude was differently affected by whole-body cold exposure when PPG was measured from the finger (peripheral tissue) and the earlobe and ear canal (core tissue), and concluded that ANS regulation is highly affected in peripheral tissue, whereas core vasculature remains almost untouched, indicating a prevalence of the body to maintain the conditions in vital organs at the expense of peripheral circulation (Budidha and Kyriacou, 2019). Alian et al. demonstrated that, when low-body negative pressure (LBNP) was used as a model of hemorrhage, both time- (Alian et al., 2011a) and frequency-domain parameters (Alian et al., 2011b) measured from the variability of PPG amplitude from the earlobe (core vasculature) and the finger (peripheral vasculature) showed different behavior after LBNP, and that peripheral vasculature showed larger changes that were not significant from core tissue, probably due to greater changes in vasoconstriction in peripheral tissue controlled by sympathetic activity.

In this study, it was observed that most PRV- and HRV-derived indices increased during cold exposure when measured from any of the locations. However, certain differences were observed. Remarkably, ear canal indices did not show a statistically significant difference due to cold exposure when any of the indices were compared among stages, while most of the other locations showed differences between baseline measurement and cold exposure, as well as between baseline measurement and cold recovery. This behavior observed from the ear canal could be a hint of the differences on vascular regulation that is performed by the ANS when the body is exposed to temperature differences (Fox, 2016). Interestingly, HRV failed to show any difference among stages when LF, HF, and TP were measured, while most of the PRV-derived indices showed differences between baseline measurement and the subsequent stages. Nonetheless, these results need to be considered with care due to the short segments used for analysis, that may affect the results obtained from frequency-domain analysis (Task Force of the European Society of Cardiology and The North American Society of Pacing and Electrophysiology, 1996).

Although the same trend was observed between data obtained from HRV and most PRV locations during the test, it is remarkable how over- and under-estimation are a constant factor in PRV analysis. Moreover, it tended to increase during cold exposure, and was higher when PRV was measured from the toe and the ear canal. These two locations could be considered as the most peripheral and the most core vasculature of the four locations used in this study, respectively, and it should be further analyzed how this differences may be influenced by ANS activity in these sites. It is also interesting to observe how the values measured during baseline were achieved from almost all locations after 10 min of recovery from the cold recovery, but how they were affected almost immediately at the beginning of the cold exposure. This could be considered as an example of the behavior of ANS regulation performed over the cardiovascular system during thermal changes.

It is important to remark that only the fiducial points that proved to detect cardiac cycles in a strongly reliable way were used for each subject and each PPG signal, and PRV data was obtained after important pre-processing stages applied to the PPG signals in order to improve their signal-to-noise ratio. Hence, these results can be considered as a strong indication of the differences between PRV and HRV when cardiovascular conditions are modified.

### 4.2. Relationship Between PRV and HRV During Whole-Body Cold Exposure

It was hypothesized that cold exposure affected the relationship between HRV and PRV, implying that PRV may not be a suitable surrogate of HRV under conditions that alter the vasculature and that it may contain different information due to cardiovascular changes.

From the Friedman rank sum test, it was observed that there were statistically significant differences between HRV and PRV, when the latter was measured from different body sites. However, the relationship between PRV and HRV changed during each stage, and from each location. Interestingly, and in line with the results obtained from the other analyses, toe and ear canal PRV consistently showed statistically significant differences to HRV. Also, frequency-domain indices, especially nLF and LF/HF, were not found different between HRV and PRV. This was probably due to the short time of analysis.

In general, the finger and the earlobe were the locations in which less differences were observed, and during all stages the earlobe proved to be the body site in which the relationship between HRV and PRV was less affected by the changes in temperature. Especially during the baseline measurement, it was observed that HRV and PRV differed especially when RMSSD, nHF, SD1, and SD1/SD2 parameters were measured. All these parameters, except for SD1/SD2, reflect the short-term HRV and PRV. Hence, PRV and HRV tend to differ more in short-term indices. In this same line, some indices showed no difference among locations. These parameters, which include LF, TP, nLF, LF/HF, and SD2, are expected to be a measurement of long-term variability (Khandoker et al., 2013). Hence, the lack of differences may be explained by the short measurement and due to the changes that are induced with short exposure to cold temperatures, that may not be reflected in long-term variability changes.

SD1, RMSSD, and HF reflect parasympathetic activity in HRV (Shaffer and Ginsberg, 2017), which usually leads to diminished heart rate and lowered force of atrial contraction, among other effects (Drew and Sinoway, 2012). In vessels, most of the ANS activity is controlled by the sympathetic nervous system, which is in charge of vasoconstriction and vasodilation in response to environmental changes (Lombard and Cowley, 2012). In this study, it was observed that a diminished temperature induced a higher similarity between these indices from HRV and PRV in different body sites, which might be explained by a lower parasympathetic activity and an increased sympathetic activity. It is not clear how sympathetic and parasympathetic changes may be affecting PRV-derived indices, and it might be possible that PRV may be affected by these changes in a different manner when compared to HRV, which is mainly a reflection of vagal activity (Laborde et al., 2017), and that sympathetic changes in vascular autonomic activity are observable from PRV indices. SD1/SD2, on the other hand, is supposed to be an index of short-term and long-term changes of ANS activity (Khandoker et al., 2013). Hence, a change in either parasympathetic or sympathetic activity should be reflected in this index, as was observed in the results obtained in this study.

However, further studies are needed to better understand and characterize PRV changes, and to evaluate how sympathetic changes may be affecting PRV-extracted indices. This could be done by using blockade techniques for assessing the contribution of each branch of the ANS to PRV indices, or by comparing PRV results to more specific measurements such as microneurography. To the knowledge of the authors, the only blockade study that has been performed to evaluate changes in PRV was done by Pellegrino et al. (2014). They showed that cardiovagal blockade induced an overestimation of HF measured from PRV; cardiac sympathetic blockade implied a moderate to high agreement between HRV and PRV in time- and frequency-domain indices; and dual blockade implied a poor accuracy and precision for normalized measures and LF/HF indices. Also, non-linear indices obtained from HRV and PRV were largely affected by both sympathetic and parasympathetic blockade. Hence, PRV and HRV can be supposed to act differently under different ANS conditions.

The correlation analysis was performed to further compare HRV and PRV. The main result was the stronger correlations observed from the earlobe and the finger, in all indices, compared to those measured from the ear canal and the toe. SD2 showed an interesting behavior: Significant correlations tended to show a lower correlation coefficient when PRV was measured from all locations during cold exposure; during baseline measurement and cold recovery, the correlation is slightly higher, indicating that the correlation of SD2 from HRV and PRV was more affected during the induced hypothermia response. Several studies have used correlation analysis to assess the relationship between HRV and PRV, some of them finding results similar to those reported in this paper. When PRV and HRV correlation was assessed in subjects at rest, earlobe and finger PRV have a good correlation to HRV indices (Shi et al., 2008; Lu et al., 2009; Bulte et al., 2011; Okkesim et al., 2016); however, certain changes in cardiovascular conditions have been found to alter the correlation between HRV and PRV, including changes due to mental stress (Giardino et al., 2002), changes in the position of the subjects (Lu et al., 2008; Gil et al., 2010), and changes in cardiovascular dynamics (Charlot et al., 2009).

Finally, from the Bland-Altman analysis, SD2, and pNN50, to a lower extent, showed the better agreement between HRV and PRV in all stages and from all body sites. This is in line with the results obtained from the correlation analysis and the Friedman's test results. However, LF/HF showed a good agreement as well, which is not reflected in the other analyses. This could be due to the short recordings which highly affect frequency-domain indices. Interestingly, some of the Bland-Altman plots derived from HRV and PRV data showed a behavior similar to what was hypothesized: The agreement is affected during cold exposure in all locations, but during cold recovery, the agreement tends to recover. Although Shin (2016) does not explain the location from which PPG signals were obtained, these results are in line with those shown by in his study, in which differences in the relationship between PRV and HRV were observed when ambient temperature increased.

Frequency-domain indices reflecting absolute powers, i.e., LF, HF, and TP, showed higher values of BAR's than any other indices, reaching BAR's above 100%. This might be an indication of the effect of short-term recordings on these indices, but further analyses should be performed to better understand how these indices may relate when extracted from PRV and HRV. Also, the toe and ear canal measurements were the ones that showed the higher differences between HRV and PRV, in all three stages. It is hard to conclude regarding the origin of these differences. Regarding the toe measurements, although the quality of the signals was the lowest, it is plausible that the higher differences were due to the measurement site: PRV has been shown to be affected by pulse transit time (PTT) variability (Gil et al., 2010), and the distance between the heart and the toe is larger than the others, implying a longer time for the pulse wave to arrive to the site of measurement and increasing the chances of cardiovascular changes that may affect PTT variability. And regarding the ear canal, these differences in agreement might be explained by the hypothesis that core vasculature is less affected by environmental changes than the other locations. Although HRV is measured directly from the heart, it could be considered as a measurement of the summation of the changes in ANS activity in the cardiovascular system as a whole, whereas the ear canal might be a reflection of more localized changes. Nevertheless, the measurement on this body site is relatively new (Budidha, 2016), and further analyses should be performed.

### 4.3. Limitations of the Study

One of the main limitations for the analysis of PRV under the exposed circumstances is the fact that PPG signals are highly affected by changes in vasculature derived from cold exposure. This represents an increased difficulty for obtaining high quality PPG signals and, therefore, for extracting reliable fiducial points from the signals. To overcome this difficulty, different signal quality indices were extracted from PPG cardiac cycles delimited by several fiducial points, i.e., systolic peaks, diastolic onsets, maximum slope points, and the point of intersection between tangent lines from the diastolic onset and the maximum slope point. It was found that the best quality of cardiac cycles was when cycles were delimited by the intersection point between the tangent lines, whereas the worst quality was obtained from the cardiac cycles delimited by systolic peaks. These results are in line with those obtained by Peng et al. (2015) and Hemon and Phillips (2016). However, the fiducial point selected for each case was different, according to the results of each signal, as recommended in the literature (Pinheiro et al., 2016). With this methodology, the probabilities of having a low-quality PRV time-series was reduced. Also, the IBI's and RRI's were manually corrected, to avoid outliers and mistakes that could affect the results.

It is important to consider as well that the sample size of this study was relatively small, and composed mainly of young and healthy subjects, that do not represent the population as a whole. Finally, a note should be made on the the short segments of signals used for the analyses performed in this study, which might affect the results, especially those obtained from frequency-domain parameters. These short recordings were selected in order to be able to compare the three stages, and to observe the differences along time. Although longer recordings are recommended, several studies have shown that short recordings of less than 10 min can be used reliably for the analysis of time-domain and nonlinear indices from HRV and PRV (Shaffer and Ginsberg, 2017).

## 5. Conclusion

From these results, it can be concluded that PRV and HRV should not be regarded as equal under all circumstances, and that hypothermia affects PRV in a different manner, not only when compared to HRV but also when compared among different body sites. PRV generally overestimates HRV indices, especially under cold exposure. Moreover, there seems to be a tendency to maintain the autonomic balance more properly in core vasculature. Although further investigation is needed, the results shown in this study serve as an indication of the effects of changes in vessel characteristics that can be observed in PRV, but are not reflected in HRV, and are promising for future research, which may aim to understand the contribution of parasympathetic and sympathetic activity in the measurement of these indices from PRV. Nonetheless, further research that aims to clarify the contribution of SNS and PNS on PRV, by using methodological considerations such as using blockade studies, are needed to better understand the results obtained in this study.

## Data Availability Statement

The datasets generated for this study are available on request to the corresponding author.

## Ethics Statement

The studies involving human participants were reviewed and approved by Senate Research Ethics Committee at City, University of London. The patients/participants provided their written informed consent to participate in this study.

## Author Contributions

EM-M, PK, and JM have identified the gaps in the literature and formed a hypothesis for using PRV as a tool for identifying differences in ANS activity in core and peripheral tissue. KB and TA recruited the volunteers, carried out the experiments, and collected the data. EM-M carried out the relevant data analysis as described in the manuscript. All authors have participated in discussing the obtained results and in writing this manuscript.

## Conflict of Interest

The authors declare that the research was conducted in the absence of any commercial or financial relationships that could be construed as a potential conflict of interest.
