# Supplementary material for: Heart Rate Variability (HRV) and Pulse Rate Variability (PRV) for the Assessment of Autonomic Responses
Source: Front Physiol. 2020 Jul 23;11:779. doi: 10.3389/fphys.2020.00779 (PMC7390908; doi:10.3389/fphys.2020.00779)
Supplement: Supplementary file 1 [file Data_Sheet_1.PDF]

Supplementary Material

1 SUPPLEMENTARY TABLES AND FIGURES

1.1 Figures

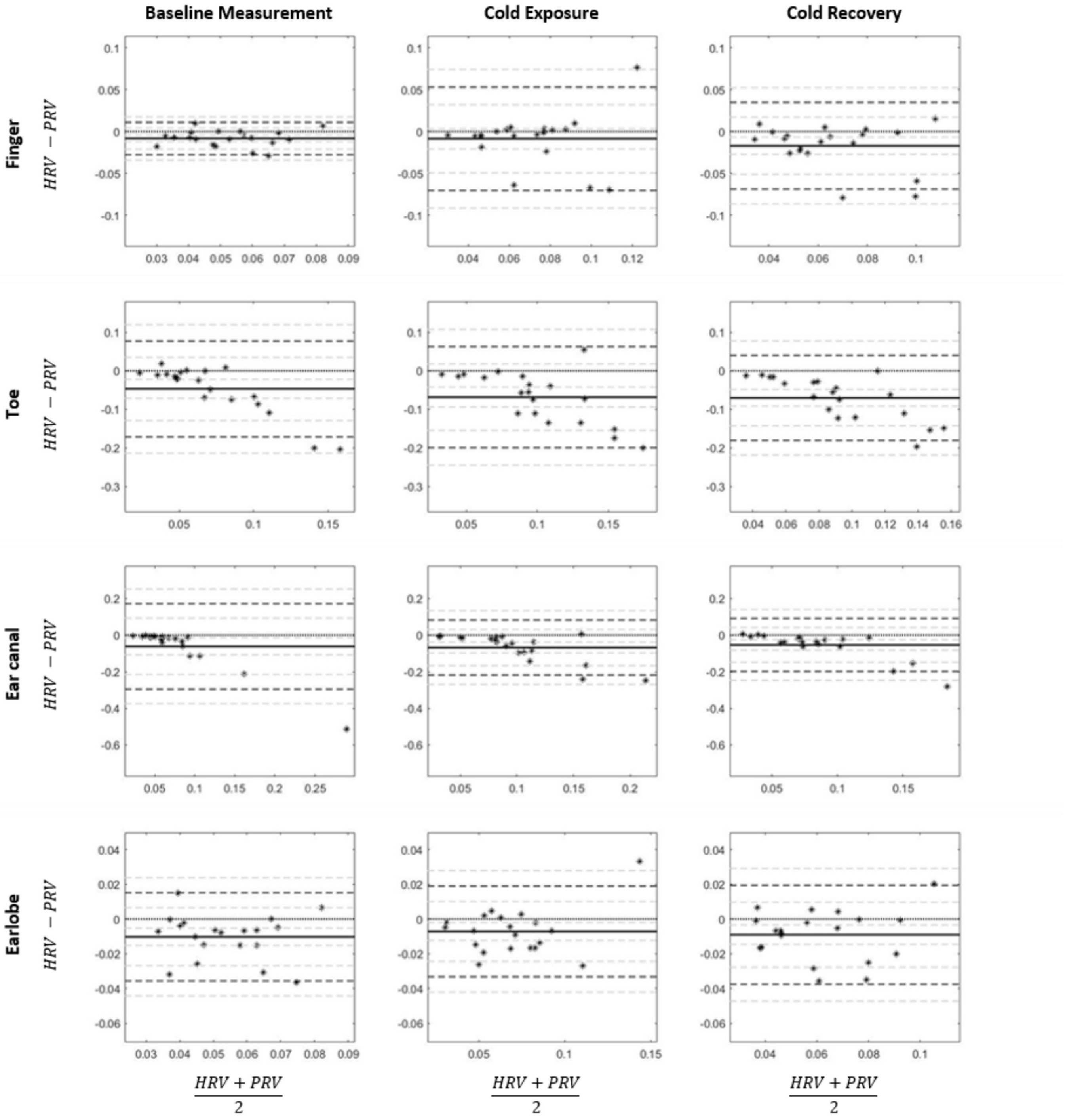

Figure S1. Bland-Altman analysis for the assessment of agreement between SDNN indices extracted from HRV and PRV, measured during the three stages of the study, from left to right: Basal measurement, cold exposure and cold recovery. From top to bottom, SDNN was obtained from the finger, the toe, the earlobe and the ear canal. Continuous line: Mean value of the difference. Dashed lines: Limits of agreement. Dotted lines: Confidence intervals. Black line: Zero difference.

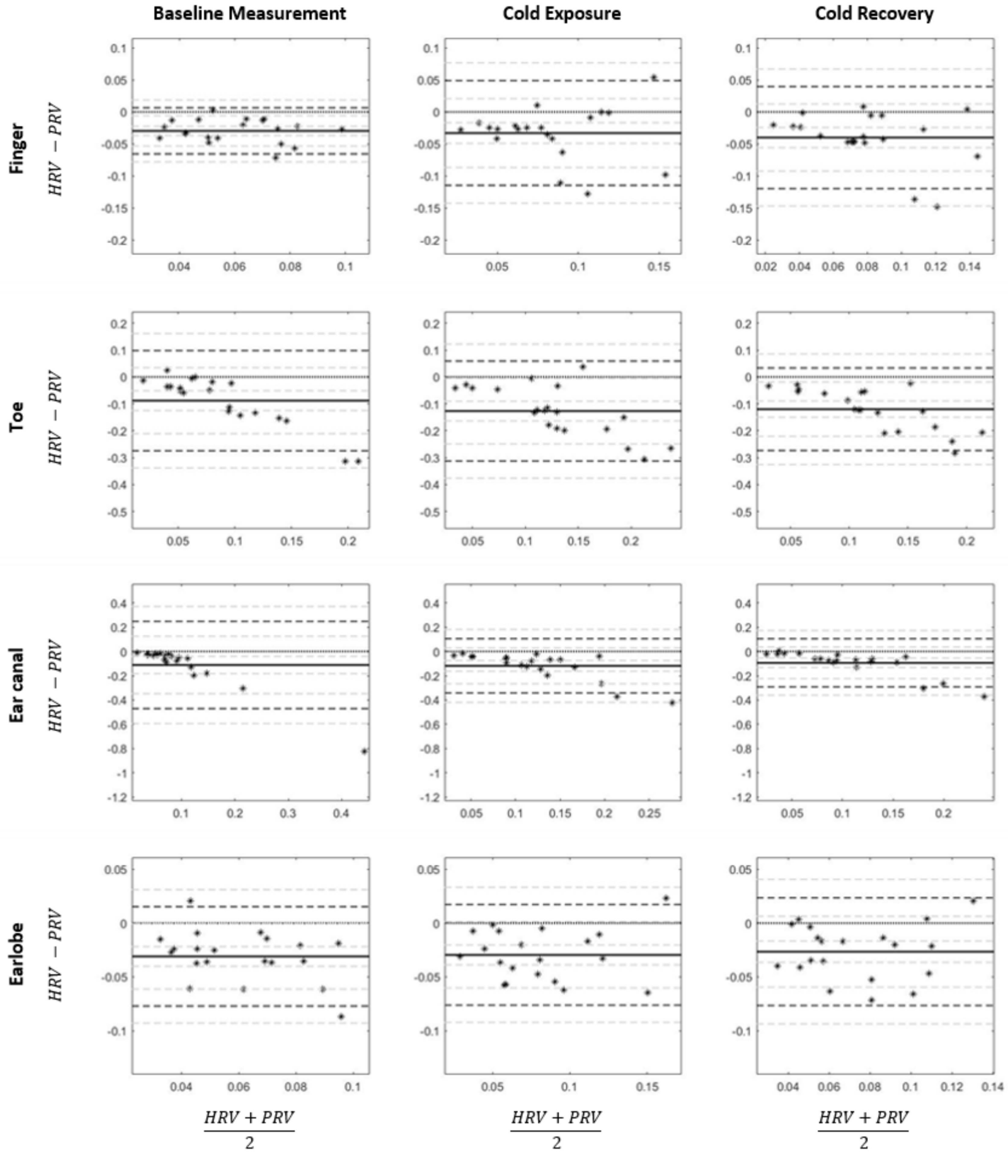

Figure S2. Bland-Altman analysis for the assessment of agreement between RMSSD indices extracted from HRV and PRV, measured during the three stages of the study, from left to right: Basal measurement, cold exposure and cold recovery. From top to bottom, RMSSD was obtained from the finger, the toe, the earlobe and the ear canal. Continuous line: Mean value of the difference. Dashed lines: Limits of agreement. Dotted lines: Confidence intervals. Black line: Zero difference.

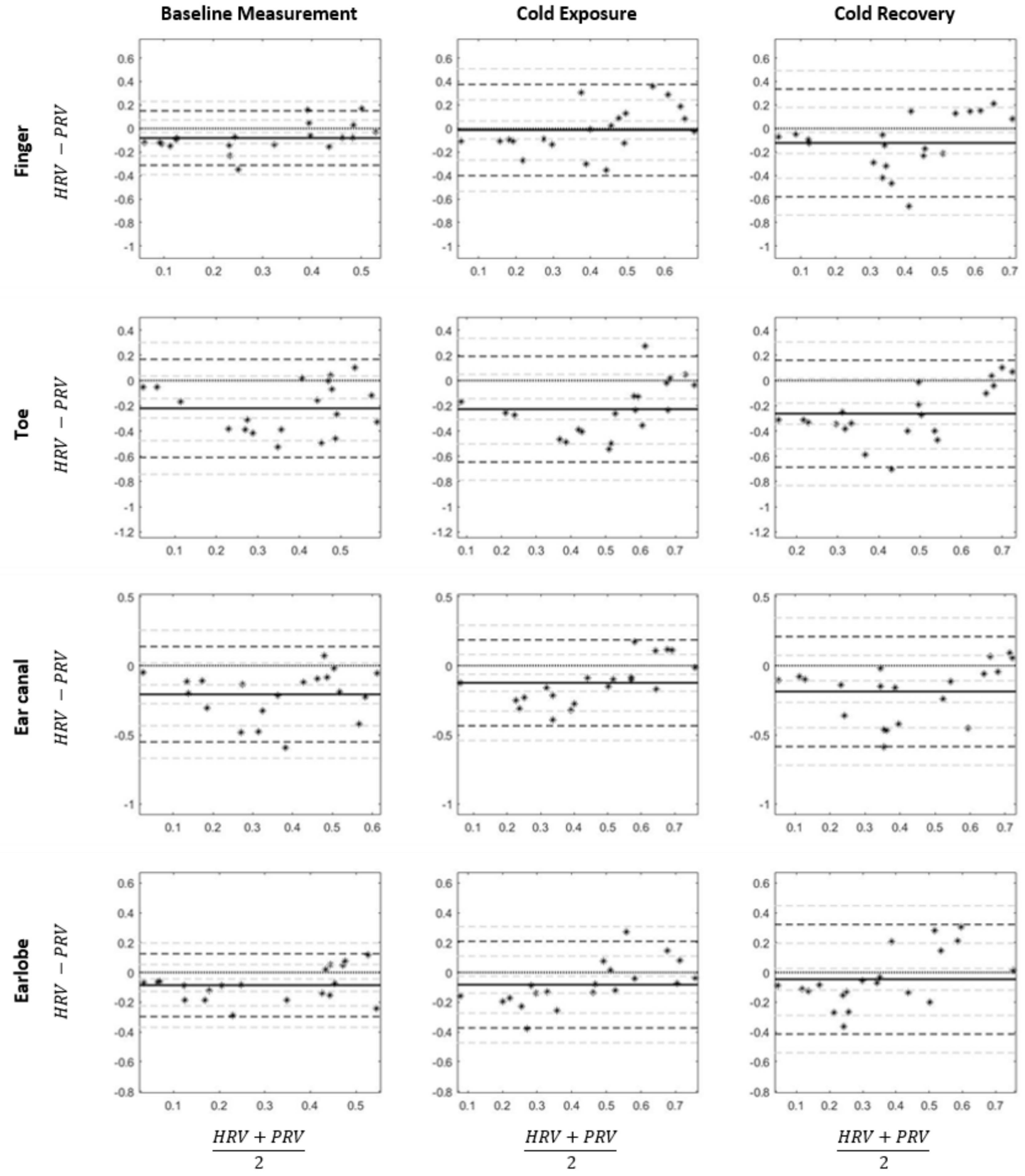

Figure S3. Bland-Altman analysis for the assessment of agreement between pNN50 indices extracted from HRV and PRV, measured during the three stages of the study, from left to right: Basal measurement, cold exposure and cold recovery. From top to bottom, pNN50 was obtained from the finger, the toe, the earlobe and the ear canal. Continuous line: Mean value of the difference. Dashed lines: Limits of agreement. Dotted lines: Confidence intervals. Black line: Zero difference.

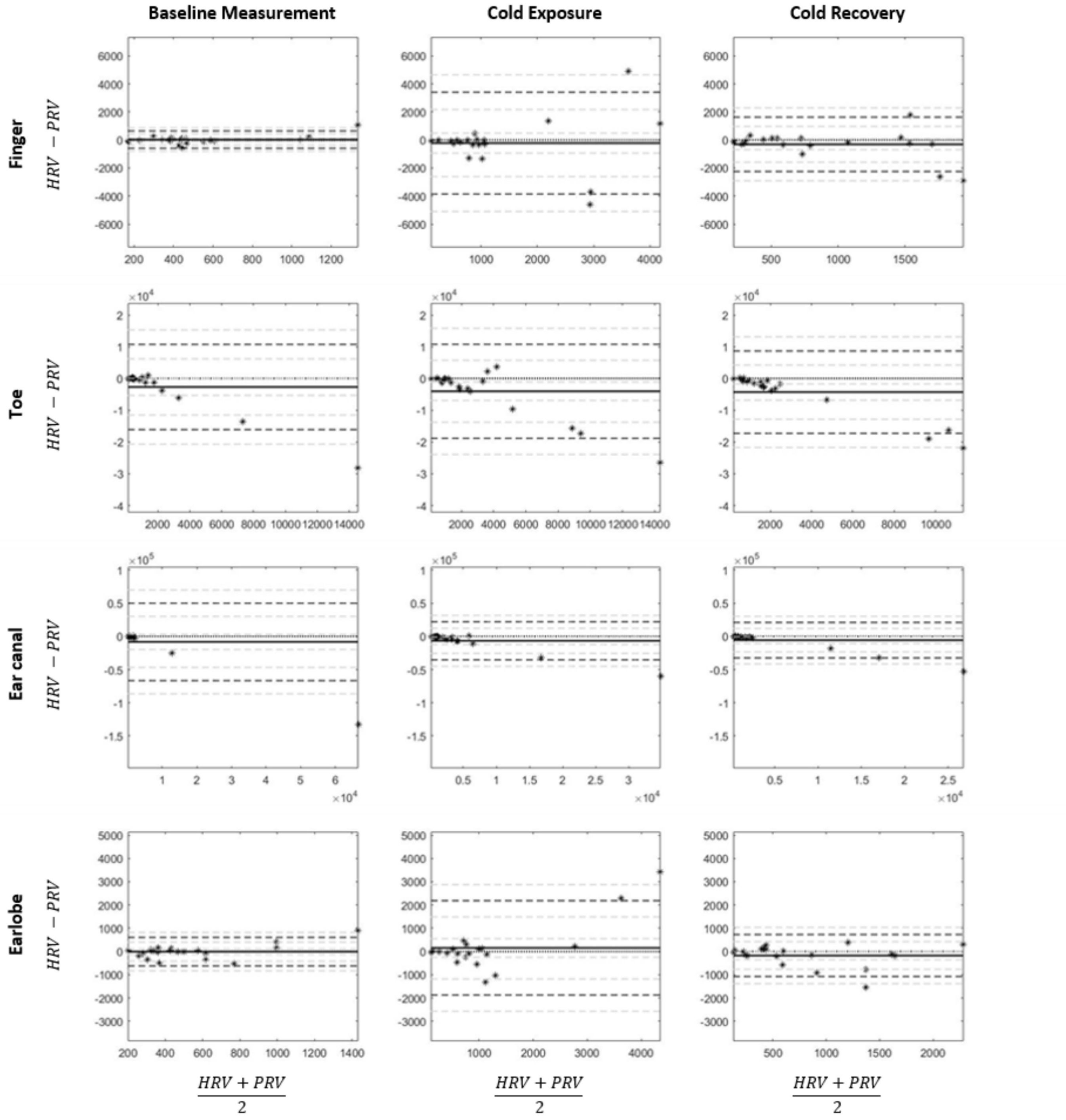

Figure S4. Bland-Altman analysis for the assessment of agreement between LF indices extracted from HRV and PRV, measured during the three stages of the study, from left to right: Basal measurement, cold exposure and cold recovery. From top to bottom, LF was obtained from the finger, the toe, the earlobe and the ear canal. Continuous line: Mean value of the difference. Dashed lines: Limits of agreement. Dotted lines: Confidence intervals. Black line: Zero difference.

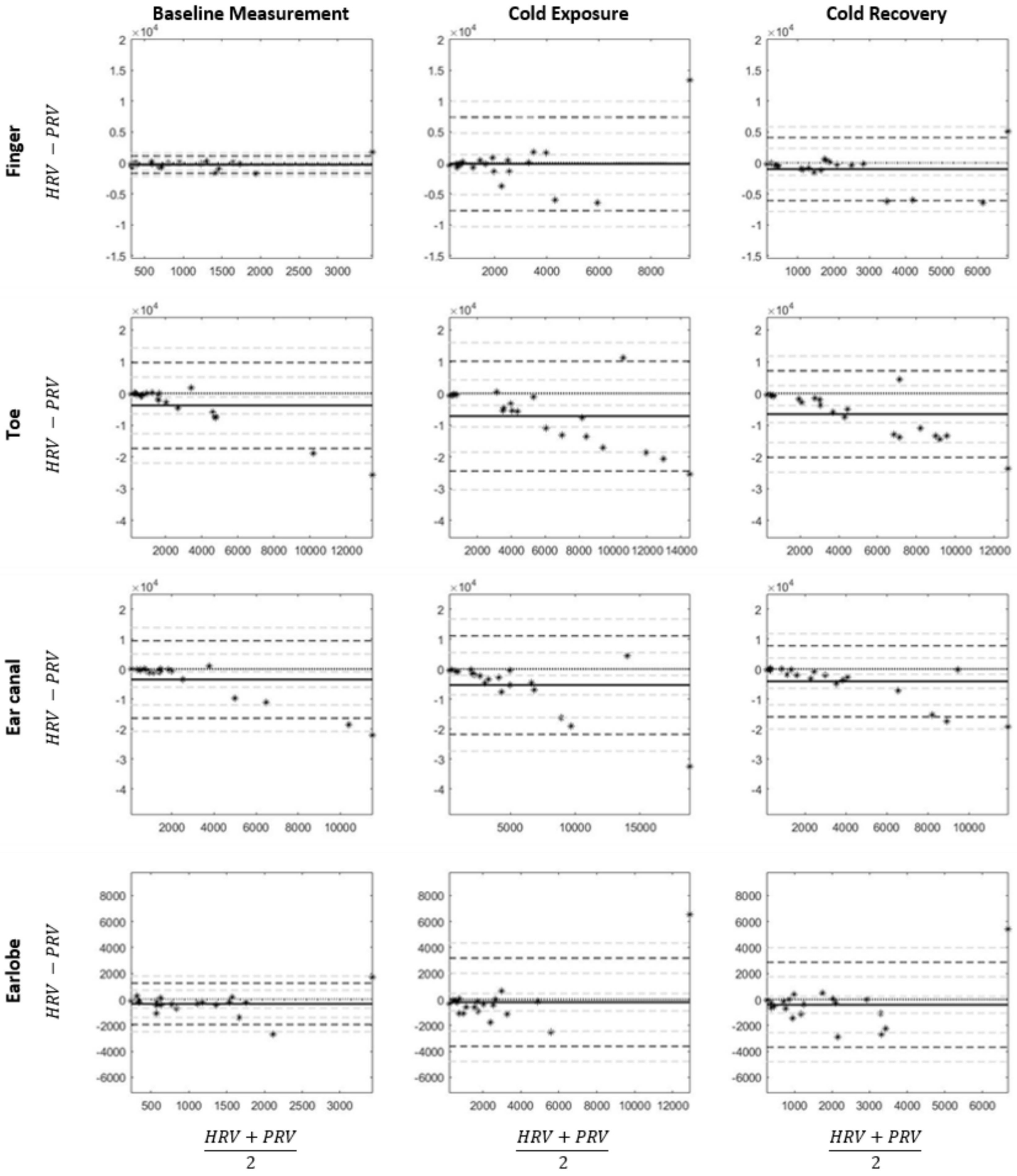

Figure S5. Bland-Altman analysis for the assessment of agreement between HF indices extracted from HRV and PRV, measured during the three stages of the study, from left to right: Basal measurement, cold exposure and cold recovery. From top to bottom, HF was obtained from the finger, the toe, the earlobe and the ear canal. Continuous line: Mean value of the difference. Dashed lines: Limits of agreement. Dotted lines: Confidence intervals. Black line: Zero difference.

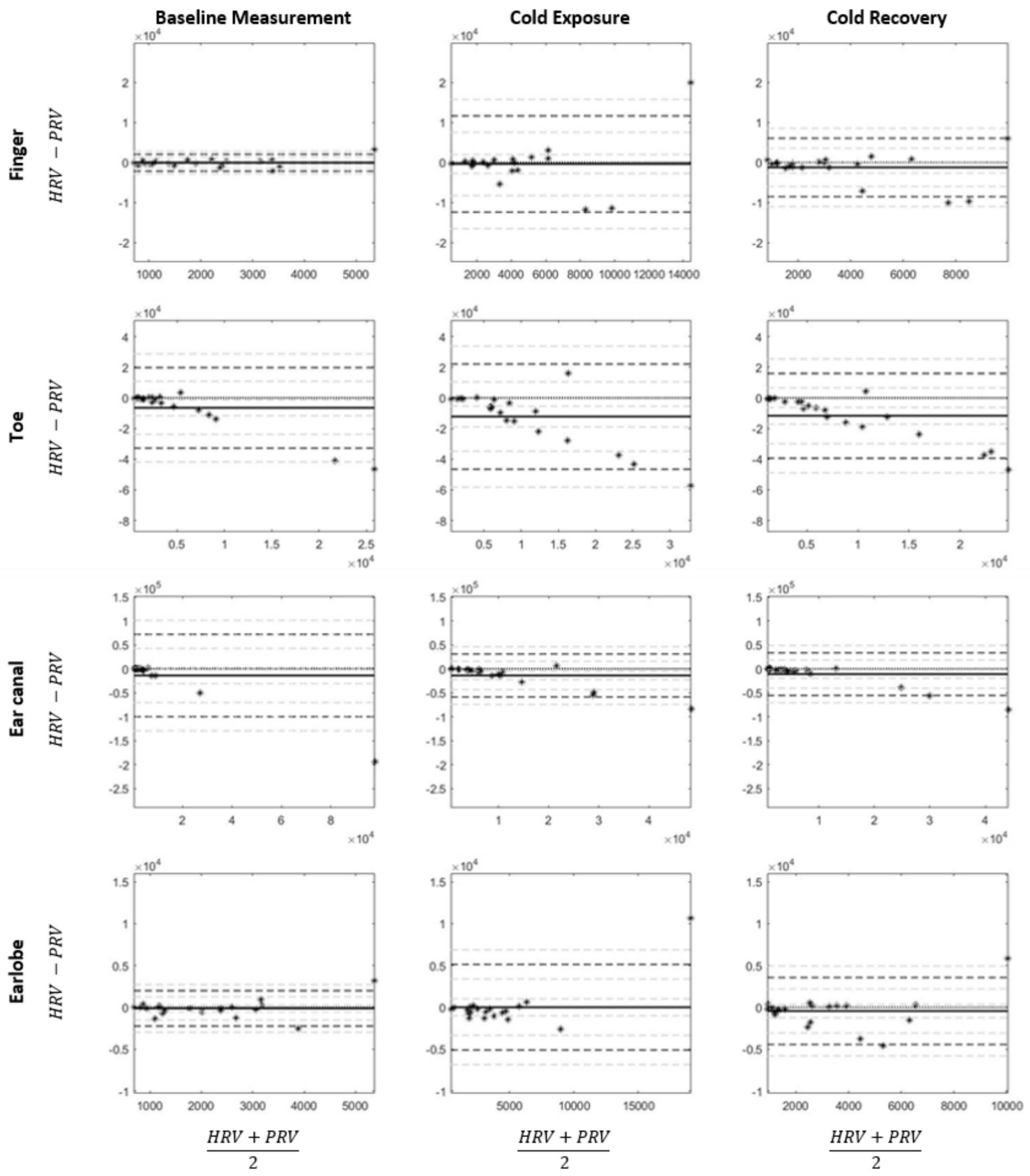

Figure S6. Bland-Altman analysis for the assessment of agreement between TP indices extracted from HRV and PRV, measured during the three stages of the study, from left to right: Basal measurement, cold exposure and cold recovery. From top to bottom, TP was obtained from the finger, the toe, the earlobe and the ear canal. Continuous line: Mean value of the difference. Dashed lines: Limits of agreement. Dotted lines: Confidence intervals. Black line: Zero difference.

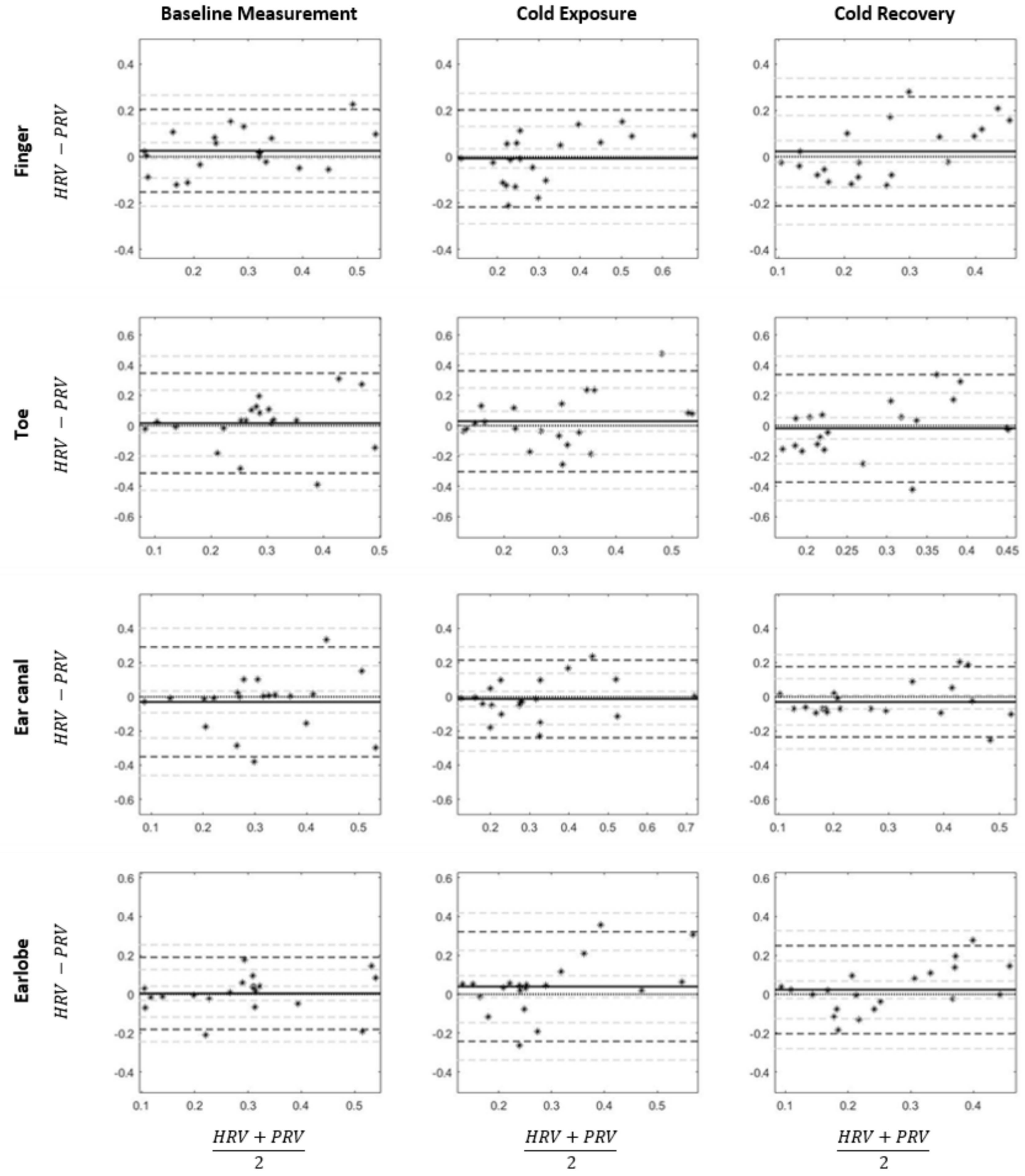

Figure S7. Bland-Altman analysis for the assessment of agreement between nLF indices extracted from HRV and PRV, measured during the three stages of the study, from left to right: Basal measurement, cold exposure and cold recovery. From top to bottom, nLF was obtained from the finger, the toe, the earlobe and the ear canal. Continuous line: Mean value of the difference. Dashed lines: Limits of agreement. Dotted lines: Confidence intervals. Black line: Zero difference.

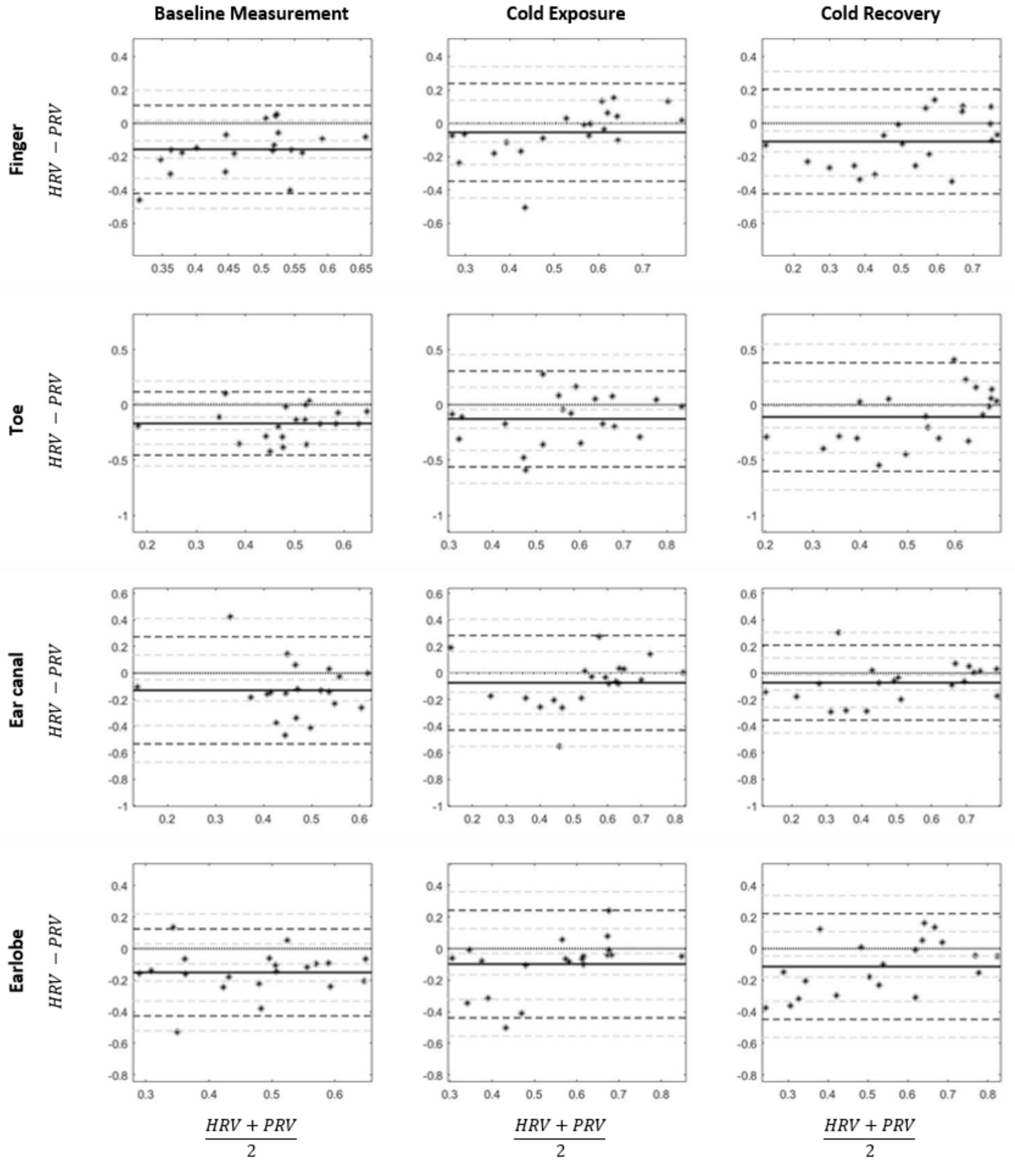

Figure S8. Bland-Altman analysis for the assessment of agreement between nHF indices extracted from HRV and PRV, measured during the three stages of the study, from left to right: Basal measurement, cold exposure and cold recovery. From top to bottom, nHF was obtained from the finger, the toe, the earlobe and the ear canal. Continuous line: Mean value of the difference. Dashed lines: Limits of agreement. Dotted lines: Confidence intervals. Black line: Zero difference.

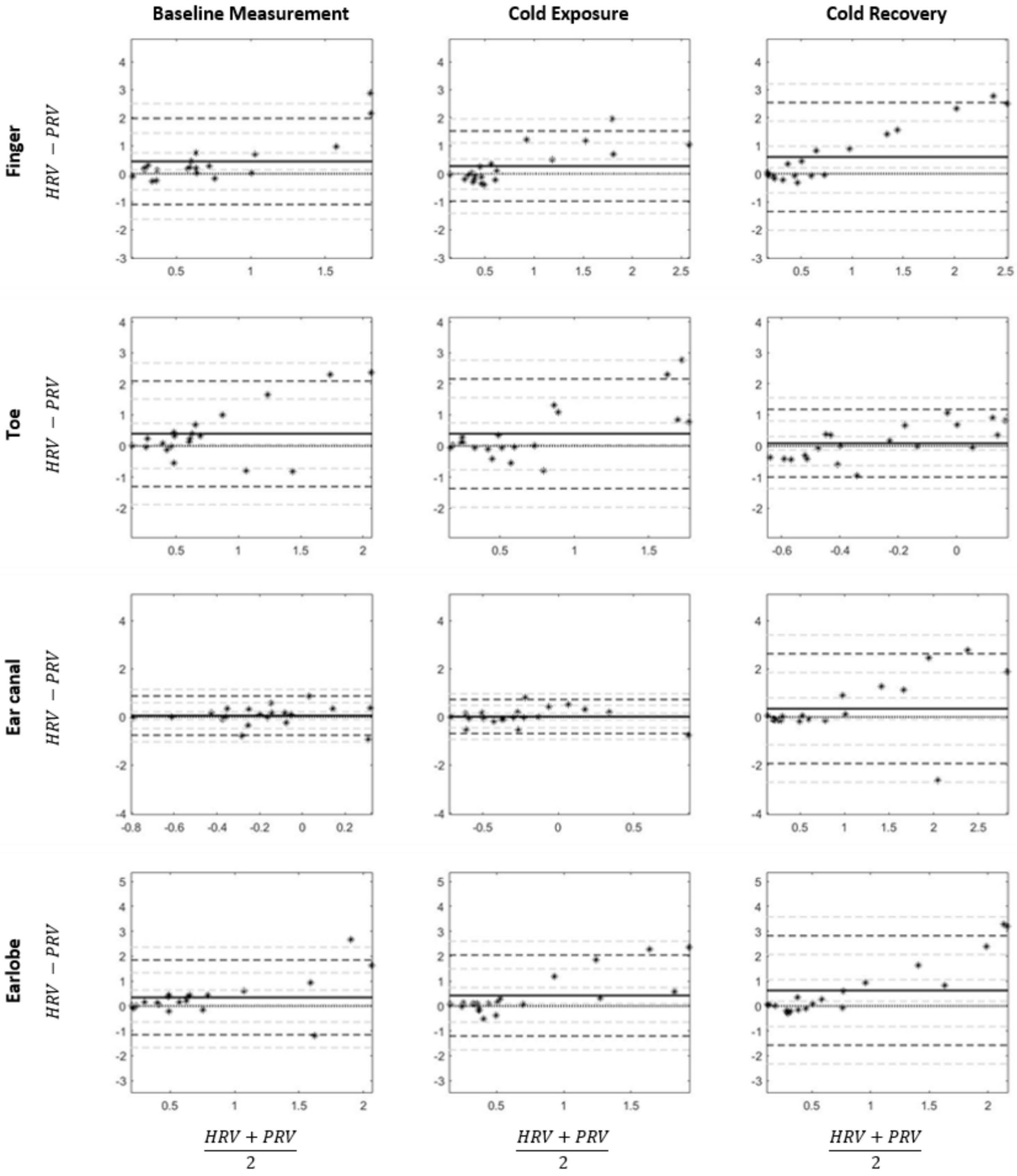

Figure S9. Bland-Altman analysis for the assessment of agreement between LF/HF indices extracted from HRV and PRV, measured during the three stages of the study, from left to right: Basal measurement, cold exposure and cold recovery. From top to bottom, LF/HF was obtained from the finger, the toe, the earlobe and the ear canal. Continuous line: Mean value of the difference. Dashed lines: Limits of agreement. Dotted lines: Confidence intervals. Black line: Zero difference.

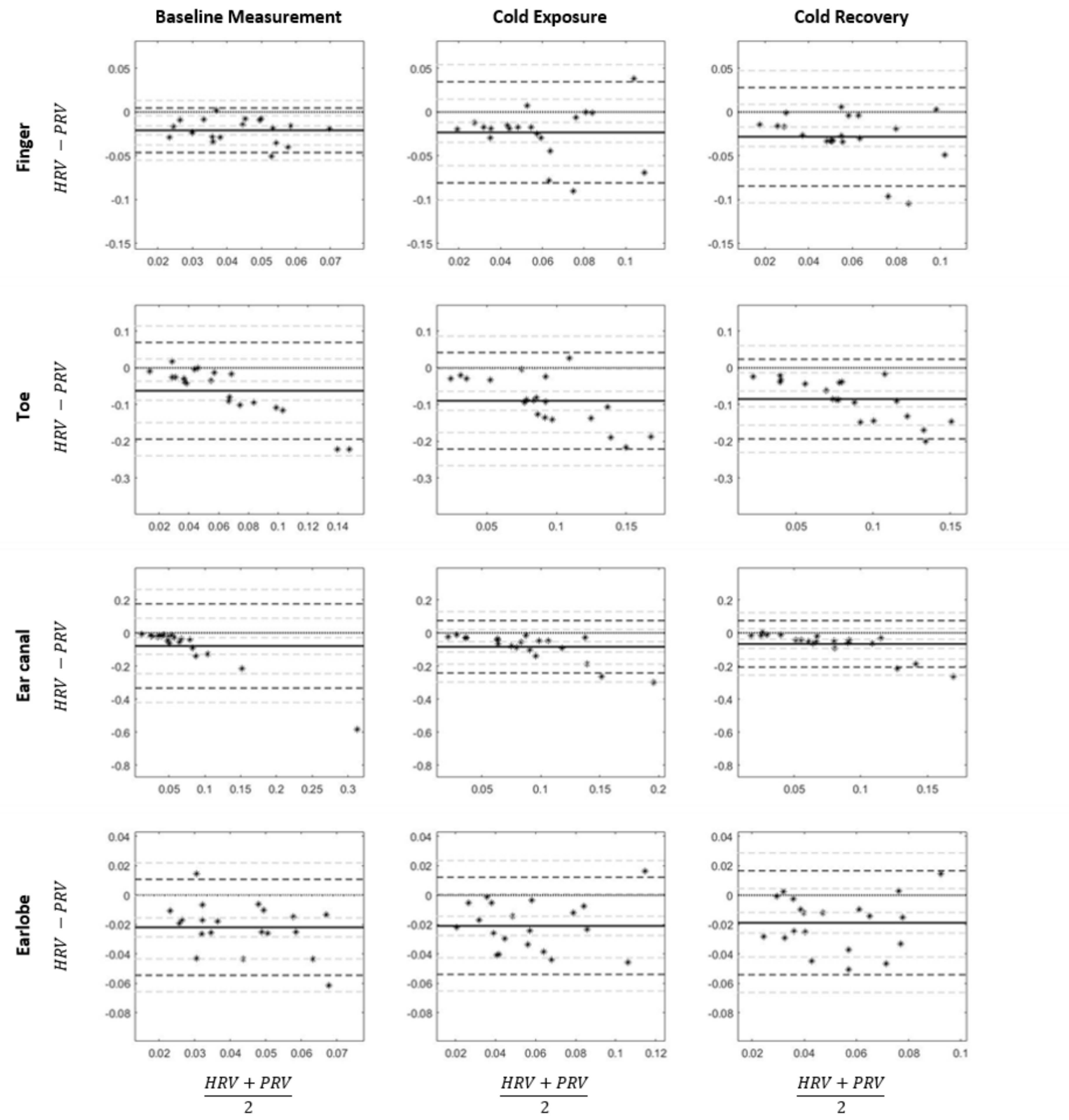

Figure S10. Bland-Altman analysis for the assessment of agreement between SD1 indices extracted from HRV and PRV, measured during the three stages of the study, from left to right: Basal measurement, cold exposure and cold recovery. From top to bottom, SD1 was obtained from the finger, the toe, the earlobe and the ear canal. Continuous line: Mean value of the difference. Dashed lines: Limits of agreement. Dotted lines: Confidence intervals. Black line: Zero difference.

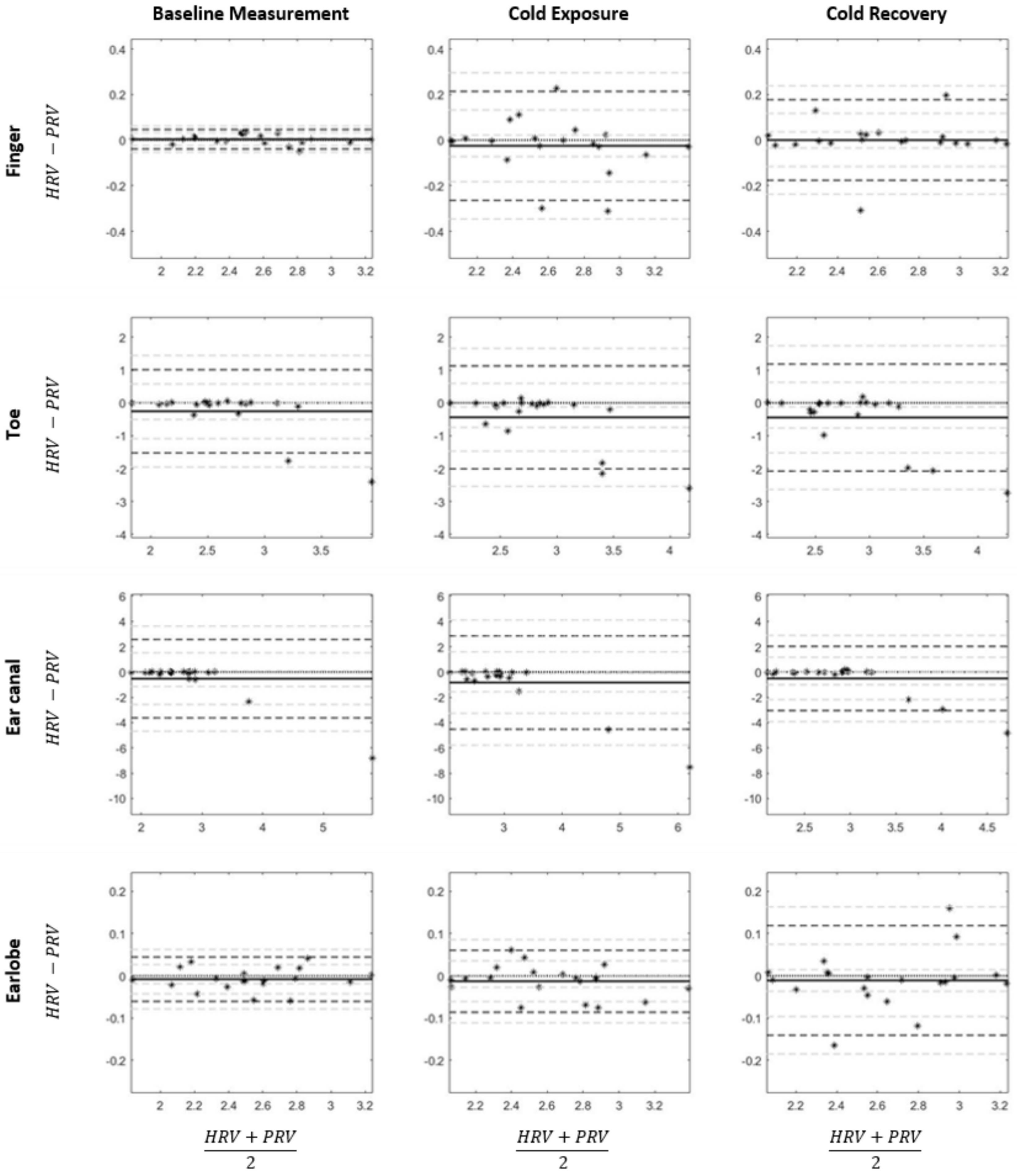

Figure S11. Bland-Altman analysis for the assessment of agreement between SD2 indices extracted from HRV and PRV, measured during the three stages of the study, from left to right: Basal measurement, cold exposure and cold recovery. From top to bottom, SD2 was obtained from the finger, the toe, the earlobe and the ear canal. Continuous line: Mean value of the difference. Dashed lines: Limits of agreement. Dotted lines: Confidence intervals. Black line: Zero difference.

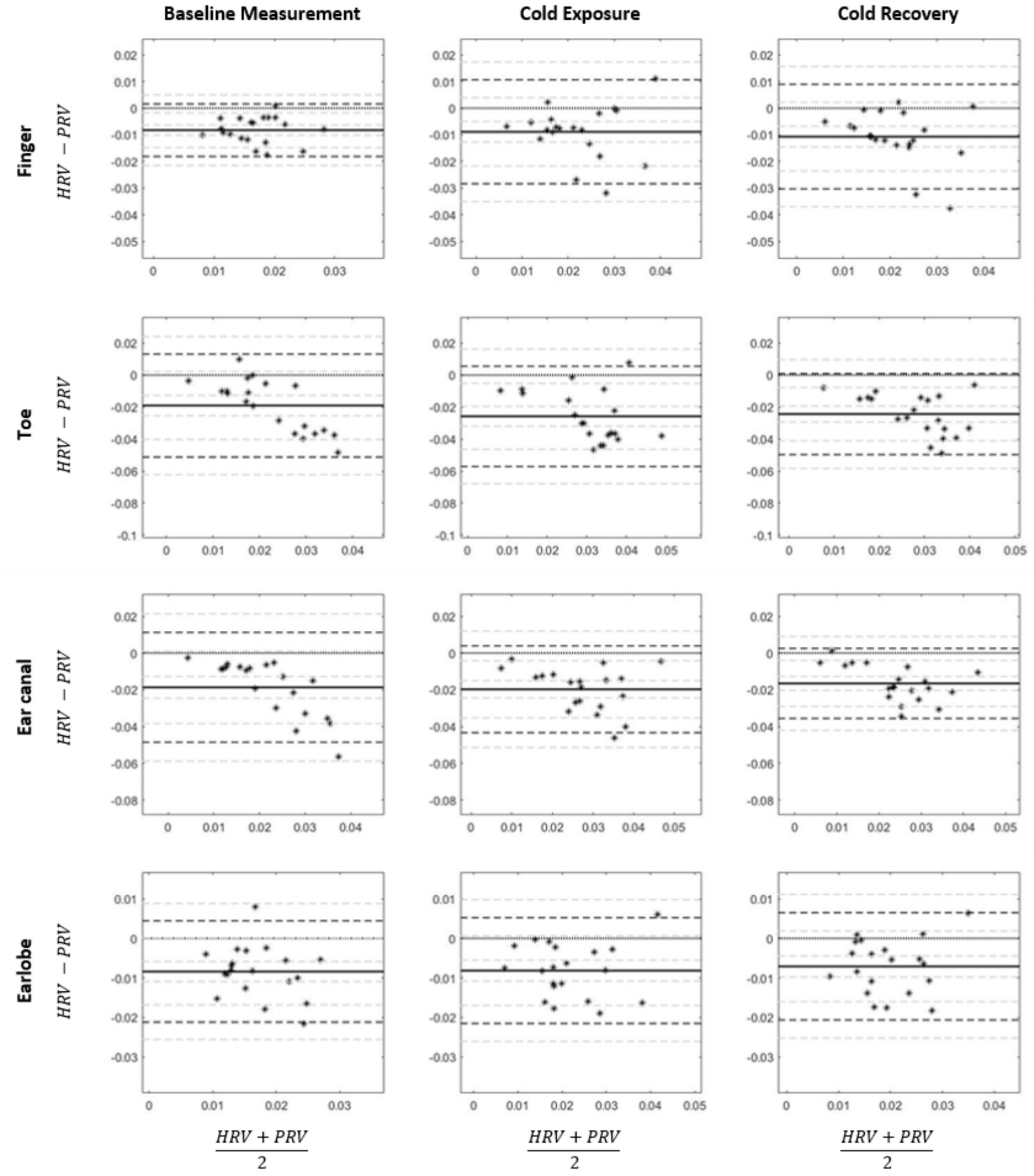

Figure S12. Bland-Altman analysis for the assessment of agreement between SD1/SD2 indices extracted from HRV and PRV, measured during the three stages of the study, from left to right: Basal measurement, cold exposure and cold recovery. From top to bottom, SD1/SD2 was obtained from the finger, the toe, the earlobe and the ear canal. Continuous line: Mean value of the difference. Dashed lines: Limits of agreement. Dotted lines: Confidence intervals. Black line: Zero difference.
